# A pressure-based force and torque prediction technique for the study of fish-like swimming

**DOI:** 10.1371/journal.pone.0189225

**Published:** 2017-12-07

**Authors:** Kelsey N. Lucas, John O. Dabiri, George V. Lauder

**Affiliations:** 1 Museum of Comparative Zoology, Harvard University, Cambridge, Massachusetts, United States of America; 2 Departments of Civil & Environmental Engineering and Mechanical Engineering, Stanford University, Stanford, California, United States of America; Coastal Carolina University, UNITED STATES

## Abstract

Many outstanding questions about the evolution and function of fish morphology are linked to swimming dynamics, and a detailed knowledge of time-varying forces and torques along the animal’s body is a key component in answering many of these questions. Yet, quantifying these forces and torques experimentally represents a major challenge that to date prevents a full understanding of fish-like swimming. Here, we develop a method for obtaining these force and torque data non-invasively using standard 2D digital particle image velocimetry in conjunction with a pressure field algorithm. We use a mechanical flapping foil apparatus to model fish-like swimming and measure forces and torques directly with a load cell, and compare these measured values to those estimated simultaneously using our pressure-based approach. We demonstrate that, when out-of-plane flows are relatively small compared to the planar flow, and when pressure effects sufficiently dominate shear effects, this technique is able to accurately reproduce the shape, magnitude, and timing of locomotor forces and torques experienced by a fish-like swimmer. We conclude by exploring of the limits of this approach and its feasibility in the study of freely-swimming fishes.

## Introduction

Fishes display a remarkable array of morphologies, which they use to play many different types of ecological roles, from grazers to apex predators. This diversity in form and function has arisen from many selective pressures, and a number of these pressures are related to locomotion [1–4]. Fish need to evade predators, capture prey, migrate, and move through complex environments. As such, many outstanding questions about the relationships between form and function in fish are biomechanically-driven. For example, understanding how body shape influences swimming specializations [5,6], how material properties of the body dictate swimming performance [7–11], and the relative contributions of individual fins to overall movement [12,13] all remain largely unresolved issues in the study of aquatic locomotion. While many approaches, including computational (e.g., [9,13]), physical modeling (e.g., [7,11,14,15]), and experimental (e.g., [12,16,17]), can be taken to answer these questions, one promising approach is to leverage a detailed understanding of the magnitudes and distributions of the time-varying forces and torques that fishes generate to effect locomotion [18–21]. Despite the value of this information for understanding biological swimming, fish evolution, and developing bioinspired underwater vehicles, the nature of a fish’s fluid environment renders direct measurements of forces and torques impractical.

Herein, we detail and characterize a technique for estimating the forces and torques acting on the body of a fish-like swimmer that uses an indirect pressure-based approach. Because of the difficulty in measuring locomotor forces and torques directly, we instead obtain the desired force and torque information through knowledge of the surrounding fluid dynamics, following in the tradition of existing strategies such as those proposed by Noca et al. [22], Gurka et al. [23], Dabiri [20], and van Oudheusden et al. [24]. The force of the fluid acting on the animal’s body can be given as Eq 1: the sum of pressure and shear (viscous) forces acting at the animal’s surface [25–28].

F(t)=−∫npdA+∫τ⋅ndA (1)

The corresponding torque is given by:

T(t)=−∫p(r×n)dA+∫r×(τ⋅n)dA (2)

Bold indicates vector or tensor quantities: **n** is the normal unit vector indicating the direction perpendicularly outward from the body, **τ** is the viscous stress tensor, **F** is the force acting on the body, **T** is the torque acting on the body, and **r** is the moment arm vector, measured from the center of rotation (center of mass, for a fish). Additionally, *t* is time, *A* describes surface area, and p is pressure in the fluid.

The simplicity of these equations has a useful consequence: when the shear forces and torques are small, total forces and torques can be estimated from the pressure terms alone. At the relatively high Reynolds numbers (Re) where many fishes operate (10,000–5,000,000 [26,27]), the shear contributions are small enough that we can still arrive at an accurate estimate of forces and torques using the pressure terms while ignoring the shear terms. Formally, Bale et al. [29] demonstrated this approximation to be reasonable for a fish-like swimmer in their decomposition of locomotor forces into components derived from viscous- and pressure-based effects.

Forces and torques could be easily approximated from pressures via Eqs 1 and 2 if pressure could be readily measured, but directly obtaining pressure measurements around a freely-moving body has also proven to be difficult. Pressure sensors can be challenging to apply and may involve animal surgery [30,31]. Additionally, large numbers of sensors are required to provide detailed flow information over the whole body [24,30–33]. More problematically, sensors and their attached cables may interfere with the fluid flow around the animal and therefore will provide, at best, approximations of the pressures around a body that occur during non-invasive study [32,34]. Recently, several methods for calculating pressure fields have been proposed (e.g., [23,24,32,35–37]). These methods rely on experimentally-measured velocity data and on the Navier-Stokes equations describing fluid motion. The equations are used to calculate pressure by integrating the pressure gradient term along paths through the velocity field (e.g., [32,35]). Note that the simplification of Eqs 1 and 2 does not imply neglect of viscosity during these pressure field calculations.

Measuring flow velocity around biological swimmers is commonly accomplished using two-dimensional digital particle image velocimetry (DPIV) (e.g., [38–40]), by tracking the motions of near-neutrally-buoyant particles in a plane illuminated by a laser light sheet [41,42]. The advantages of the 2D approach include the ease with which the position of the animal can be tracked and the flow patterns can be visualized and interpreted, as well as the limited effects on animal behavior and relative ease in convincing the animals to swim with their whole bodies in a light sheet [38–40,42]. While taking a 2D approach to measuring velocity is highly desirable, it also introduces the assumption inherent to 2D DPIV analysis—that the flow perpendicular to the imaging plane is limited [42].

Thus, we focus here on validating this approach, i.e., calculating forces and torques from a thin plane of measured velocity data and the corresponding pressure field. To do this, we used a physical model to produce empirical, biologically-relevant flows. In this system, we can measure forces and torques using a load cell, while simultaneously applying our proposed pressure-based technique to estimate these same forces and torques. We then performed a quantitative comparison of the measured and estimated values to identify the conditions where our assumptions—first, that pressure effects dominate shear effects, and second, that most of the fluid’s velocity is captured in the horizontal imaging plane—are met for fish-like swimmers, and reasonable estimates of forces and torques can be produced.

In the second section, we describe the physical model of fish-like swimming and the measurement of forces and torques. We also describe the pressure-based calculations used to estimate these same values. In the third section, we describe and compare the measured and estimated forces and torques. In the final section, we discuss the conditions where the measurements and estimates match, and the utility and limitations of this approach for the study of biological locomotion.

## Methods

### Simultaneous data collection

#### Foil design

**Fig 1:**
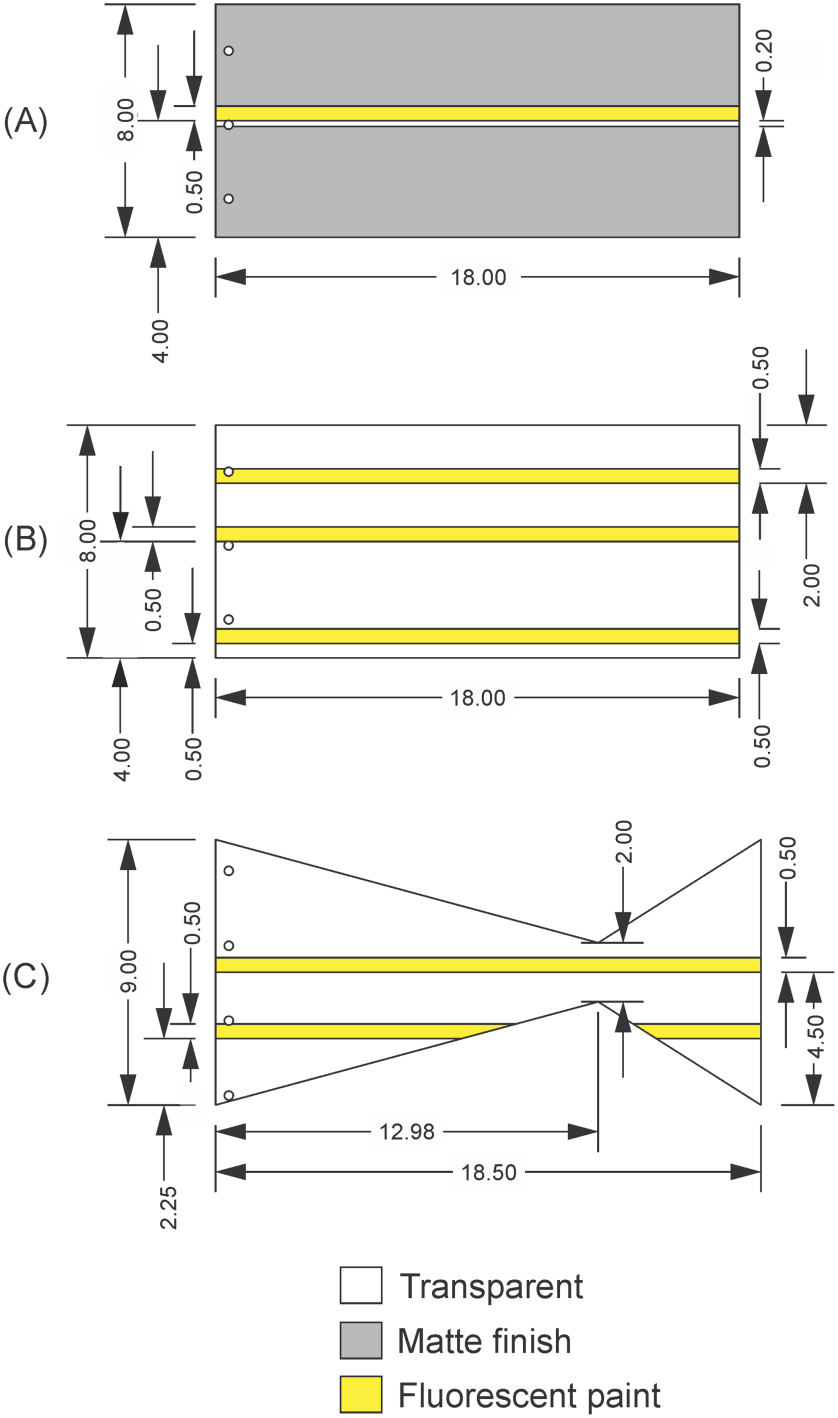
Schematics of experimental foils. (A) Rectangular foil. (B) Rectangular foil for 3D testing. (C) Tail-shaped foil. All dimensions are given in centimeters.

The principal foil design was based on the “3_3” rectangular foil from Lucas et al. [11], where three layers of transmatte plastic shim stock (ARTUS Corp, Englewood, NJ, USA) were bonded with transparent epoxy. This created a foil of flexural stiffness EI = 5.5x10^-5^ Nm^2^. Two versions of this foil were constructed. In the first (Fig 1A), prior to construction, the transmatte finish was removed from a narrow strip at the foil’s midline, using 90% ethanol, on each of the three layers of plastic, creating a transparent window. After construction, a thin strip of fluorescent paint was added just above this window. In the second version (Fig 1B), all of the transmatte finish was removed from each layer of plastic, and three strips of fluorescent paint were added. All of these modifications facilitated imaging.

In addition, one foil of a more complex shape was constructed, based on Feilich and Lauder’s [43] Shape 2. This tail-shaped foil featured a narrow caudal peduncle and a triangular tail (Fig 1C). This foil was crafted from 1 mm thick clear plastic shim stock (ARTUS Corp, Englewood, NJ, USA) with a flexural stiffness EI = 6.91x10^-5^ Nm^2^ [15]. Strips of fluorescent paint were applied to this foil at the midline and displaced 2.25 cm downward from the midline, so as to cross the gap between the body and tail regions of the foil (Fig 1C).

The flexural stiffnesses of all three foils were within the range values found in fish [7].

#### Flapping-foil system

**Fig 2:**
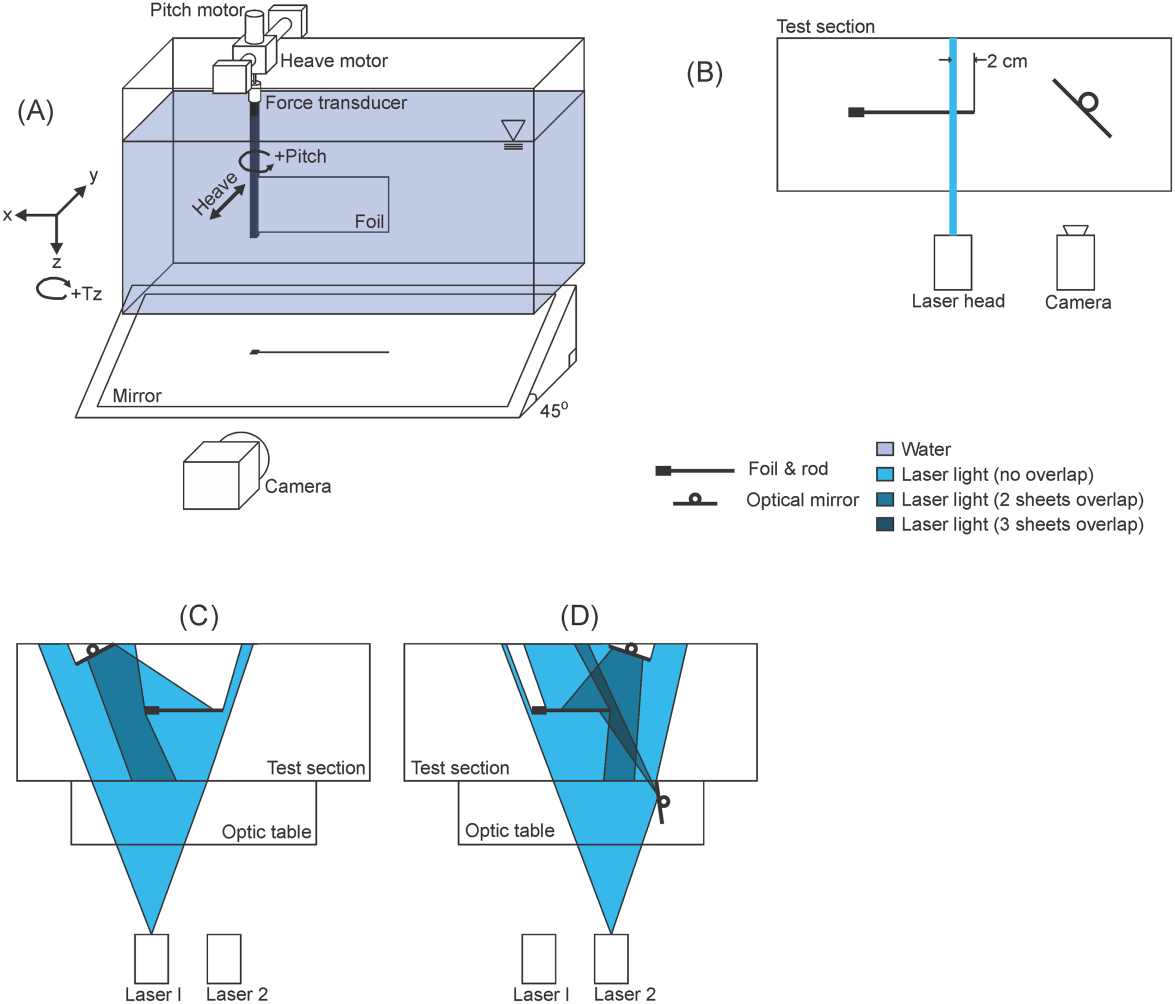
Schematics of testing systems. (A) Mechanical flapping foil testing apparatus in isometric view. (B) Overhead view of the transverse imaging setup. (C) Overhead view of the light sheet path of Laser 1. (D) Overhead view of the light sheet path of Laser 2. Laser 1 and Laser 2 were used simultaneously.

Foils were actuated into oncoming flows using the mechanical flapping-foil system described by Lucas et al. [11] and depicted in Fig 2A. In brief, the leading edge of the foil was clamped by a rod formed by two aluminum spars with rectangular cross-section, each having a chord length of 10 mm and thickness 1.5 mm. An ATI Nano-17 six-axis force-torque sensor (model SI-50-0.50, ATI Industrial Automation, Apex, NC, USA; resolution: forces = 1/80 N, torques = 1/16 Nm) attached to the rod enabled measurements in the X (streamwise), Y (lateral), and Z (vertical) directions. This assembly was suspended in a recirculating flume by a carriage on top of the tank. A set of heave and pitch motors on this carriage actuated the foil. A suite of custom LabVIEW (National Instruments Corp., Austin, TX, USA) programs were used to control foil motion and collect position and force and torque data. Each experimental trial was conducted twice: once with the foil and the rod, and once with only the rod.

#### Imaging systems

Flow was rendered visible by seeding the flow tank with near-neutrally-buoyant (density 1060 kg/m^3^), VESTOSINT 1164 white nylon 12 particles with an average diameter of 50 μm (Degussa Corporation, Piscataway, NJ, USA; now Evonik Industries AG, Essen, GER). These particles and the foil were illuminated with light sheets generated by a continuous wave laser (either Coherent 10W argon-ion or OptoEngine 532A solid-state lasers).

To allow automated kinematics detection during later analyses, the illuminated foils needed to be in high contrast with the dark background. In addition, the force and torque estimation method required pressure information from both sides of the foil. Therefore, the flow visualizations could not have any shadows, i.e., places where flow information would be missing. To this end, a set of three mirrors was used in conjunction with the foils’ transparent strips and fluorescent paint (Figs 1 and 2) to reflect the laser light and illuminate each foil and the entirety of the surrounding water. Two small, rectangular mirrors were fitted with adjustable mirror mounts, and each was attached to the end of a shaft. These “shaft mirrors” were suspended from the far side of the flow tank: one upstream of the foil, and one downstream (Fig 2C and 2D). These mirrors were positioned at an angle to the wall of the tank so as to each reflect light back toward the foil (Fig 2C and 2D). The final mirror was positioned on a stage between the incoming laser and the flow tank (Fig 2D).

The lighting scheme worked as follows. The first laser sheet was aimed at the bottom edge of the foil’s fluorescent paint (Fig 2C), causing the foil to be the brightest object in the field of view, in contrast to a black background. This first laser sheet also illuminated the flow upstream of the foil and was reflected off the upstream shaft mirror to eliminate the shadow from the foil’s rod (Fig 2C). The second laser sheet was aimed through the window in the foil (Fig 2D), about 1 mm below the first sheet. This illuminated the flow on the far side of the foil (Fig 2D). This laser sheet also was reflected onto the fluorescent paint at the foil’s trailing edge, by both the downstream shaft mirror and the mirror outside the tank (Fig 2D). The reflections from these mirrors aided in illuminating the trailing edge of the foil, which tended to curve so as to create shadows.

Because surface waves created glare which distorted images of particle motion and introduced error into foil tracking, a set of baffles lined with black foam were placed at the water surface upstream and downstream of the rod, effectively removing the free surface. The baffles suppressed the surface waves, thereby removing glare and ensuring a uniform, dark background that contrasted with the bright foil.

Ventral views of the flow tank were filmed at 1000 frames per second using a high-speed camera (Photron PCI-1024; 1024x1024 resolution, 17 μm pixel size) and a 45° mirror positioned below the tank (Fig 2A). The camera was activated upon receiving a LabVIEW pulse trigger, which allowed these data to be collected simultaneously with position, force, and torque data. In all cases, data were collected for the duration of three complete motion cycles.

During investigations of 3D effects, vertical flow magnitudes were also measured. An OptoEngine 532A solid-state laser light sheet was projected vertically to illuminate the foil and surrounding flow in a transverse section (Fig 2B). The laser was positioned so as to intersect each foil 2 cm upstream of its trailing edge. A Photron Fastcam Mini AX50 (1024x1024 pixel resolution, 20 μm pixel size) high speed camera collected video at 1000 frames per second off of a single 45° mirror located downstream of the foil (Fig 2B) for three replicate motion cycles.

#### Types of tests conducted and actuation parameters

Each test that involved motion of a foil was conducted twice, once in each of two motion programs. The first program, a heaving program, actuated the foil in sinusoidal, lateral heaving motions without introducing pitch. This program has been used extensively in previous study of foil locomotion [11,14,44–46]. Moreover, we expected that the relatively large degree of flow separation at the foil’s leading edge induced by this program would reveal the sensitivity of the force estimation method to the presence of complex flow structures around the body.

The second program featured 0° angle of attack motion [11]. Here, the pitch angle of the foil’s leading edge was continuously changed as the foil heaved laterally so as to maintain a constant 0° geometric angle of attack into the oncoming flow. Compared to the heaving program, this program has been demonstrated to lead to more fish-like kinematics [11,15] and improved swimming performance, in fact, to near-maximum [11,47]. To account for the rotation of the force-torque sensor’s axes during pitch, the measured force values (*F*_*x*,*meas*_ and *F*_*y*,*meas*_) were resolved into streamwise (*F*_*x*_) and lateral (*F*_*y*_) components using Eqs 3 and 4, where θ was the instantaneous pitch angle in radians [11].

$$F_{x}=F_{x,meas}\;\text{cos}\;\theta +F_{y,meas}\;\text{sin}\;\theta$$ (3)

$$F_{y}=-F_{x,meas}\;\text{sin}\;\theta +F_{y,meas}\;\text{cos}\;\theta$$ (4)

In each motion program, several types of tests were conducted so as to explore the limits of pressure-based force and torque estimation. The first of these tests was the dynamic test, which was conducted with the rectangular foil with one paint strip/window (Fig 1A). In these tests, the foil’s leading edge heave amplitude and the flow speed were fixed at 1.5 cm and 30 cm/s, respectively, and the flapping frequency was ramped up from 0.5 Hz to 2.5 Hz in 0.5 Hz steps. These frequencies are within the range of those used by the caudal fin of a fish, with the upper limit constrained by the capabilities of the flapping-foil system [16,48,49]. The foils therefore swam in accelerating, decelerating, and steady conditions, at Re = 54,000 and in a Strouhal number (St) range 0.06–0.53, where Re was based on foil chord-length, and St was based on peak-to-peak trailing edge amplitude. This test was designed to reveal how sensitive the force calculation was to transient flows when velocity information was gathered at the foil midline, where three-dimensional effects are expected to be minimal.

The second test, the 3D test, was conducted using the rectangular foil with three strips of paint (Fig 1B) and the tail-shaped foil (Fig 1C). Here, the heave amplitude, actuation frequency, and the flow speed were all fixed, to 1.5 cm, 1.5 Hz, and 30 cm/s, respectively, leading to an Re range of 54,000–55,500 and St range of 0.15–0.43, where again Re was based on foil chord-length, and St was based on peak-to-peak trailing edge amplitude. Initially, the light sheet was positioned at the midline as in the dynamic tests described above. Then, the light sheet was shifted vertically so as to illuminate, in turn, the each of the other paint strips. We anticipated that these locations would experience different degrees of out-of-plane flow due to increasing edge effects. Moreover, the tail-shaped foil could experience complex, interacting flows between the “body” and “tail” regions. Thus, this test would reveal how sensitive the force calculations were to deviations from 2D flow. In addition, the transverse light sheet imaging scheme was used to quantify how strong the vertical flows were around both foils.

The third test, the static test, was conducted with the rectangular foil with one paint strip/window (Fig 1A), 0° pitch, and flow speeds 10, 30, and 50 cm/s (Re = 18,000, 54,000, and 90,000, respectively). Unlike in the previous tests, a deliberately misaligned force-torque sensor was used, as the near-zero magnitude of the lateral forces expected here would be within the sensor’s alignment error (±0.01 N). By misaligning the sensor, larger “lateral” force magnitudes would be registered (and slightly smaller “streamwise” forces), and these could be resolved back into the true streamwise and lateral forces. This was accomplished using Eqs 3 and 4, while setting the pitch angle to a constant 0°. This test would demonstrate how the technique would break down when shear effects become substantial relative to pressure effects.

For each test, data were collected for three replicate motion cycles. In the static case where no motion cycle was defined, three one-second replicates were collected. In all cases, the force-torque sensor’s sampling rate was 1000 Hz.

### Flapping foil data processing

The flapping-foil system generated time-series for pitch angle, heave amplitude, force, and torque during each trial conducted. Since the pressure-based calculations were designed to yield forces and torques acting on the foil, excluding the rod, a comparison could not be made directly to the measurements from the flapping-foil apparatus, which could only measure from a rod-foil assembly, or from the rod alone. So, to make an appropriate comparison, the forces and torques from a given rod-only trial needed to be subtracted from the measured values from the corresponding rod-foil assembly trial. This would isolate the forces and torques acting on the foil. While the combination of the rod and foil may not be strictly linear, the approximation produced by this subtraction represents the expected effect of the rod, which is a low-frequency bias due to its inertia. A custom Python (version 2.7.11, Python Software Foundation, https://www.python.org) script was used to this subtraction in preparation for comparison with the calculated estimates, yielding time-series of forces and torques due to solely the foil.

**Fig 3:**
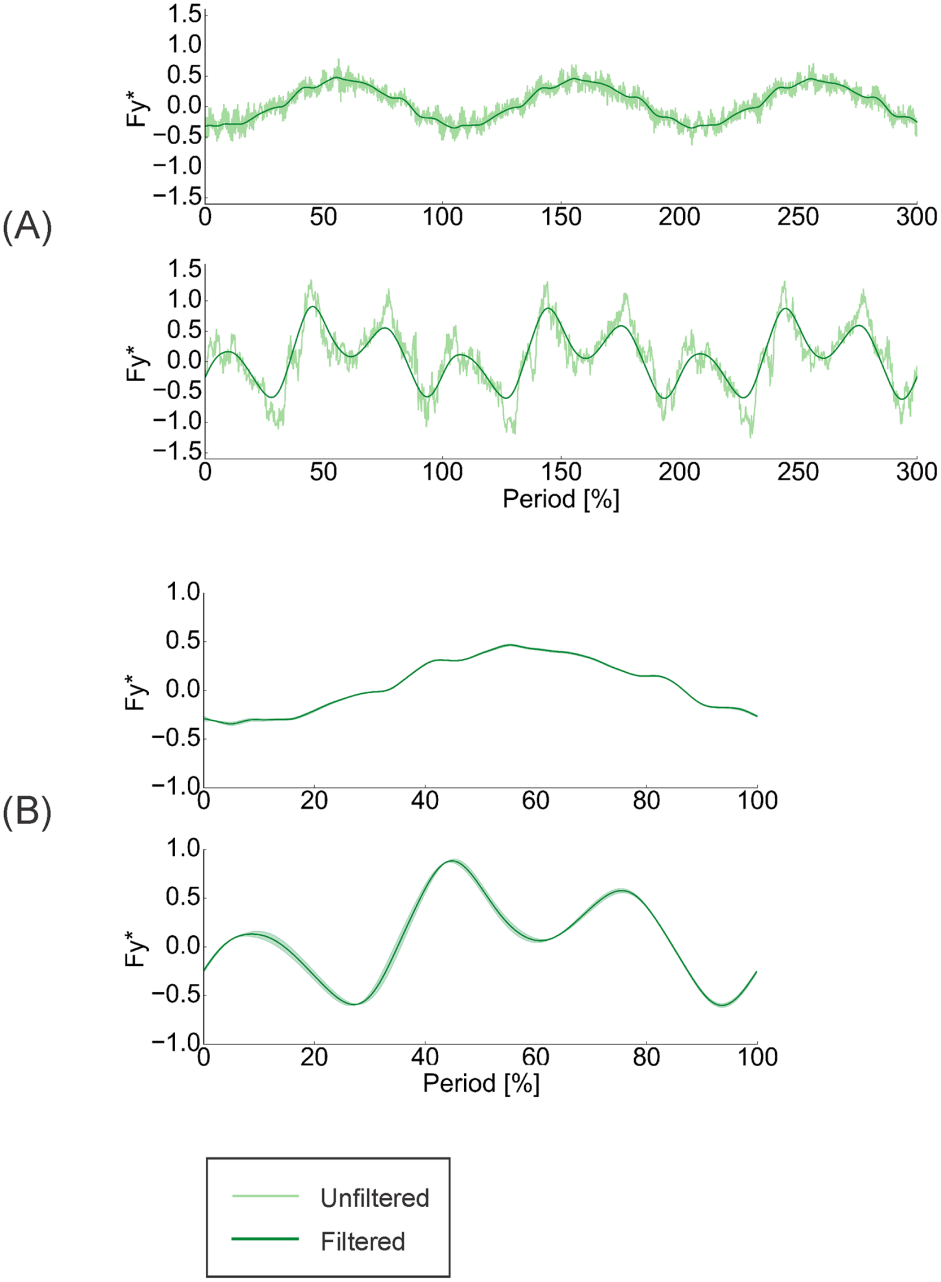
Processing of highly repeatable measured force and torque data. Measured force data were highly repeatable. (A) Example raw and filtered lateral force (*F*_*y*_) traces, taken during dynamic testing. Three motion cycles during 1.0 Hz (top) and 2.0 Hz (bottom) 0° angle of attack motions are shown. (B) Filtered, phase-averaged traces of the data from (A). Silhouettes represent standard deviations. Streamwise forces (*F*_*x*_) and vertical torques (*T*_*z*_) followed similar trends to those displayed here.

The script also applied a second-order Butterworth low-pass filter to the force and torque data from the transducer. The filter was applied in two passes, to eliminate phase shifts, with a desired cutoff frequency of 7 Hz (adjusted to 8.73 Hz in each pass to account for multiple passes) [50]. This cutoff frequency was chosen through visual inspection as a compromise between following the main trends and eliminating high frequency noise in the signals (e.g., Fig 3A).

Finally, the script performed phase-averaging of the three motion cycles, which demonstrated that there was high repeatability in the measured force signals (Fig 3B).

All forces and torques were nondimensionalized using the following equations, where * indicates a nondimensional term, *F* represents force, *T* represents torque, *ρ* is the density of fresh water, *c* is foil chord, *s* is foil span, and *v* is the flow velocity.

$$F^{*}=\frac{F}{\rho scv^{2}}$$ (5)

$$T^{*}=\frac{T}{\rho sc^{2}v^{2}}$$ (6)

### Video data processing

#### Masking foils in video data

**Fig 4:**
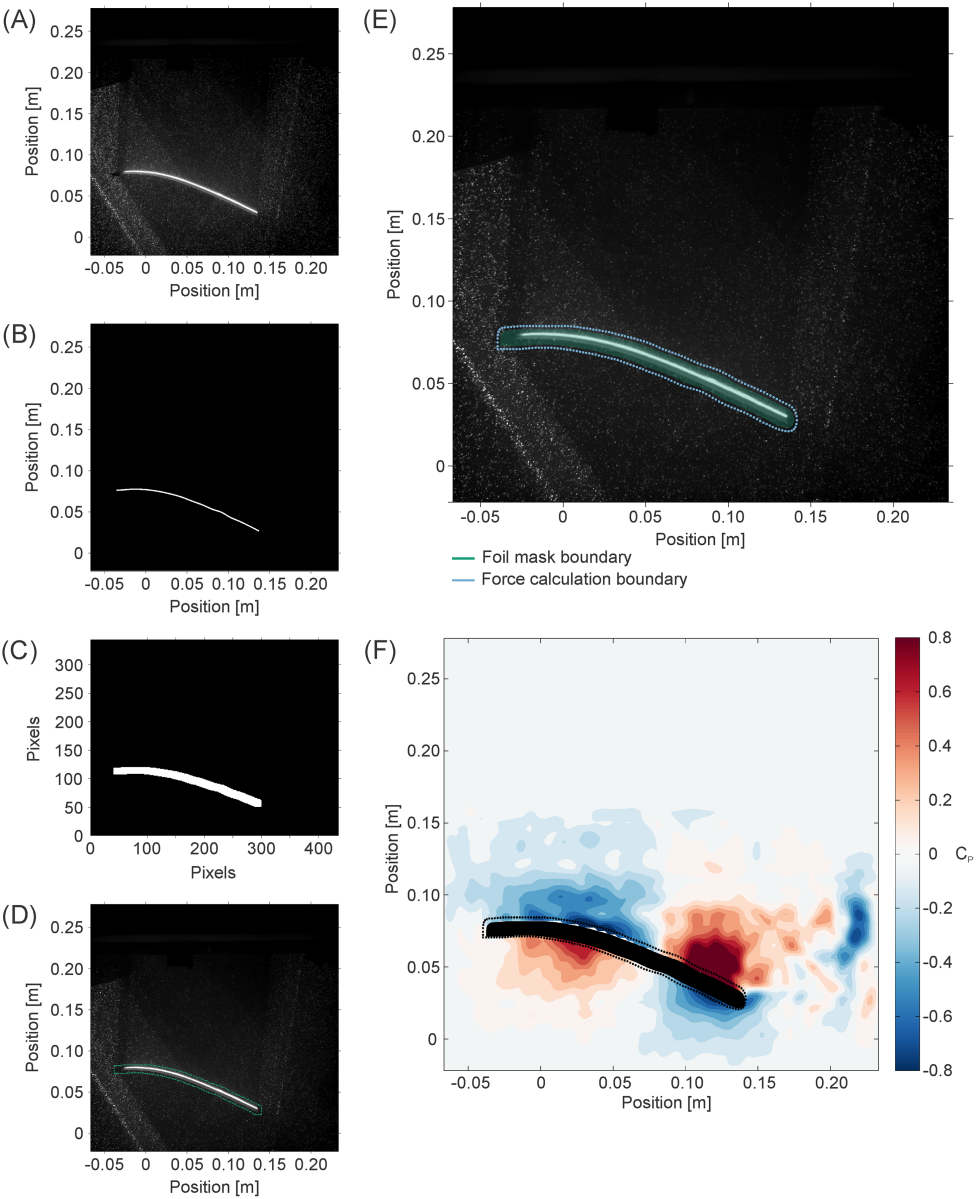
Image processing steps in making foil masks and force calculation boundaries. Boundary coordinates for foil masks and force calculation were generated using binary image processing. (A)-(D) illustrate mask generation, and the same process was used to produce force calculation boundaries. (A) A frame extracted from video of foil motion. Fluorescent paint at the foil’s midline appears as a bright line, and the portion of the foil below the light sheet is visible due to parallax of 3D structures. (B) The automatically-detected midline of the foil. (C) Binary image dilation widened the detected midline. (D) 200 equally-spaced points on the black-white boundary in (C) were extracted to use as a mask enclosing both the foil’s midline and the portion of the foil visible below the light sheet. The points depicted here were smoothed to remove jagged edges. (E) Smoothed foil mask plotted as a silhouette, and the 200-point force calculation boundary produced by the same process. (F) Pressure contour for the video frame, with the foil mask and force calculation boundary drawn in black.

A custom LabVIEW program [11] was used to automatically detect the foil as the largest, brightest object in each video frame. Because the black rod did not contrast with the background, the portion of the foil sandwiched by the 1-cm-wide rod was simulated as a 1-cm straight line extending from the leading edge of the detected foil [11]. The automatically-detected kinematics were then converted into mask boundaries, which would enclose vectors in the velocity field to indicate to the pressure-algorithm the presence of a solid object. The mask needed to be large enough to enclose the portion of the foil and rod below the light sheet which blocked the view of flow within a few millimeters of the foil due to parallax effects (Fig 4A). Mask-generation was accomplished in Matlab 2013b (MathWorks, Inc., Natick, MA, USA) by plotting the detected foil as a white line on a black field (Fig 4B), and using binary image dilation to widen the line by 1.1 δ_99_ on each side, where δ_99_ is the 99% boundary layer thickness (also called the shear-layer thickness). Boundary layer thickness was calculated using the equation below, where *x* is foil chord length and *Re* is the Reynolds number [51].

$$\frac{\delta_{99}}{x}=\frac{5}{\sqrt{Re}}$$ (7)

This dilation was enough to enclose velocity vectors inside the foil (Fig 4C). After the dilation, 200 equally-spaced coordinates on the black-white boundary were identified (Fig 4D). These were smoothed with a 5-point-span moving average filter to generate the final mask (shaded area in Fig 4E and 4F).

#### Particle image velocimetry

DPIV analysis was conducted using DaVis 8.2.2 (LaVision GmbH, Goettingen, GER). Any visible walls of the flow tank were masked. The cross-correlation analysis was conducted in multiple passes with decreasing interrogation window sizes (32x32 and 16x16) and 50% overlap. Two passes were made at each window size. During post-processing, vectors were deleted if their correlation value was <0.8, though in some rare extremes, the cutoff was set at 0.6. The empty spaces were filled by interpolation, and simple 3x3 smoothing was applied to the result, leading to a 128x128 grid of vectors. The use of this smoothing regime was motivated by Wang et al.’s [52] findings. In their analysis of error level in pressure fields calculated from velocity fields with known levels of Gaussian noise, they demonstrated that this average smoothing of velocity fields reduced the noise level in pressure fields produced by the Dabiri et al. [35] algorithm by 30–67% [52]. The flow velocity vector fields were exported for use with the pressure algorithm.

#### Nondimensionalization of vertical flow velocities

The transverse DPIV fields revealed the vertical flow magnitudes (*V*_*z*_) immediately to the right of the foil as it approached direction reversal. To provide a measure of how important vertical flows were relative to the horizontal (in-plane) flows at the same location, *V*_*z*_ was normalized to a non-dimensional metric *V*_z_*,

$${V_{z}}^{*}=\frac{V_{z}}{V_{tot}}$$ (8)

which represented the proportion of the total velocity magnitude at a given location that was in the vertical direction. By assessing the *relative* importance of vertical flow, we would be able to translate our analyses to other flows where the overall flow magnitude is different. Total velocity magnitude (*V*_*tot*_) was calculated using streamwise (*V*_*x*_) and lateral (*V*_*y*_) velocities from the DPIV taken in the horizontal plane and vertical (*V*_*z*_) velocities from the transverse plane. This calculation was performed at every point of intersection of horizontal and vertical light sheets, i.e., three positions on the rectangular foil’s span, two positions on the tail-shaped foil’s span, as indicated by the bottom edge of the fluorescent paint strips in Fig 1. Means and standard deviations of *V*_z_* from three replicate motion cycles were taken to provide a metric of repeatability.

#### Pressure-field algorithm

Pressure fields were calculated using the Dabiri et al. [35] queen2 algorithm, which was selected for its ability to handle the substantial body deformations characteristic of fish-like swimming. In their paper, Dabiri et al. [35] described extensively the function and performance of this pressure-field algorithm. In brief, the algorithm performs a direct integration of the pressure gradient term of the Navier-Stokes equations along several paths through the field. To reduce the effects of errors from individual paths, a median-polling scheme is used to choose the estimate of pressure at every point in space. Unlike other methods such as that developed by Gurka et al. [23], where a boundary value problem (i.e. the pressure Poisson equation) is additionally solved to determine the pressure (i.e. Eq 2 of their paper), the Dabiri et al. [35] approach only involves integration of the Navier-Stokes equations and not solution of the pressure Poisson equation. Dabiri et al. [35] validated their approach against computational simulations of flow around a square cylinder and an anguilliform swimmer. While Wang et al. [52] suggested that the Dabiri et al. [35] method favors speed over accuracy compared to some other methods, the validations provided by Dabiri et al. [35] and the subsequent experimental applications of the algorithm [53,54] indicate that it produces sufficiently accurate fields to be useful in experimental studies.

#### Selection of time-step for pressure calculation

**Fig 5:**
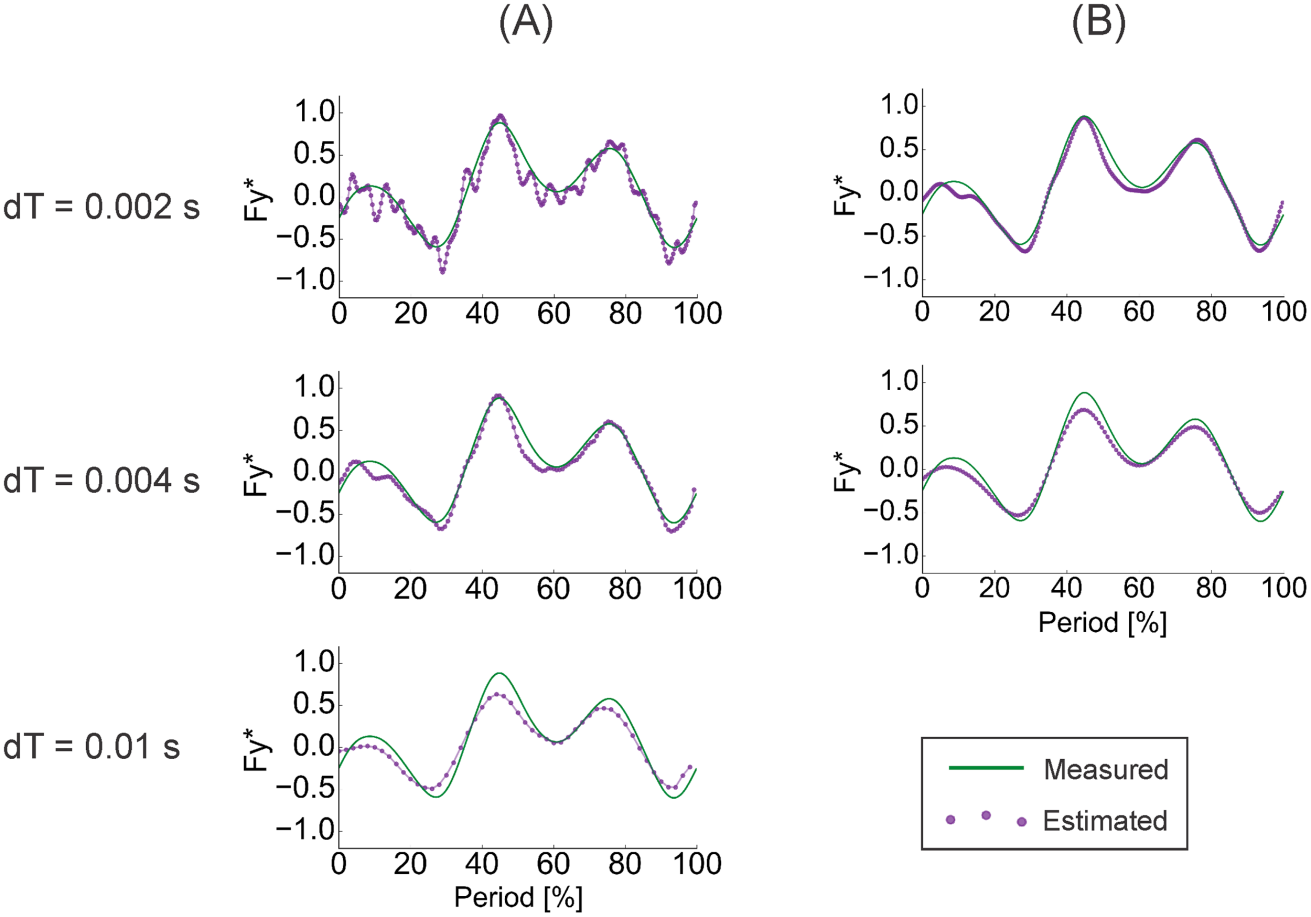
Time step selection for pressure field calculation. A comparison of the measured and calculated lateral force (*F*_*y*_) values when the rectangular foil was operating in 0° angle of attack motions at 2.0 Hz actuation frequency under different time-steps. (A) The noise in the force trace from pressure-based force calculations decreased as time step (dT) increased. (B) When a low-pass filter was applied to the noisy time traces, nearly identical traces resulted, and these traces resembled the trace produced the time step was 0.01s. Streamwise forces (*F*_*x*_) and vertical torques (*T*_*z*_) followed similar trends to those displayed here.

As the pressure-field algorithm reads in velocity data at some time interval (ex: once every 0.01 s), a larger time step would be desirable to decrease computation times. To determine what maximum time step between successive images would be permissible for high accuracy results, multiple time steps– 0.01s, 0.004s, and 0.002s –were assessed for their viability (Fig 5). Forces and torques were calculated using the pressure-based technique (following the Methods in the next section) for an arbitrarily-selected test case, the dynamic test’s 2.0 Hz trial in 0° angle of attack motions. Smaller time steps permitted more high-frequency fluctuations into the traces (Fig 5A). Because true trends can be revealed in measured data by using low-pass filtering to eliminate high-frequency effects that are more sensitive to experimental error (see “Flapping foil data processing” section), the same low-pass filter used on the sensor data was applied to the traces calculated at 0.004s and 0.002s time steps. When plotted side-by-side, the filtered traces resembled the trace produced with the 0.01s time step (Fig 5B). We confirmed that the 0.01s time step was preserving the main trends in force and torque using a Fast Fourier Transform analysis. Because the 0.01s yielded a reasonable time trace while significantly reducing computational time, this value was selected for all subsequent force and torque calculations.

#### Pressure-based force and torque calculation

Pressure-based force and torque calculation was conducted based on the first terms in Eqs 1 and 2 in Matlab 2013b. Total force and torque were found as the sum of the force and torque acting on a 200-point loop around the masked foil (Fig 4E and 4F). This force-calculation loop was generated using the same binary image dilation procedure as was used for the foil masks. A slightly larger dilation (discussed in the next paragraph) than before was required to ensure that the calculation points were located where pressure was defined (undefined within the mask) (Fig 4F). To calculate forces, pressure and the normal unit vector at each of 200 dilated boundary points were noted, and the area term was calculated as the distance between boundary coordinates times the span of the foil at the current boundary point. For torque, the moment arm was defined as the perpendicular distances from the foil’s leading edge. Total forces and torques were nondimensionalized using Eqs 5 and 6.

**Fig 6:**
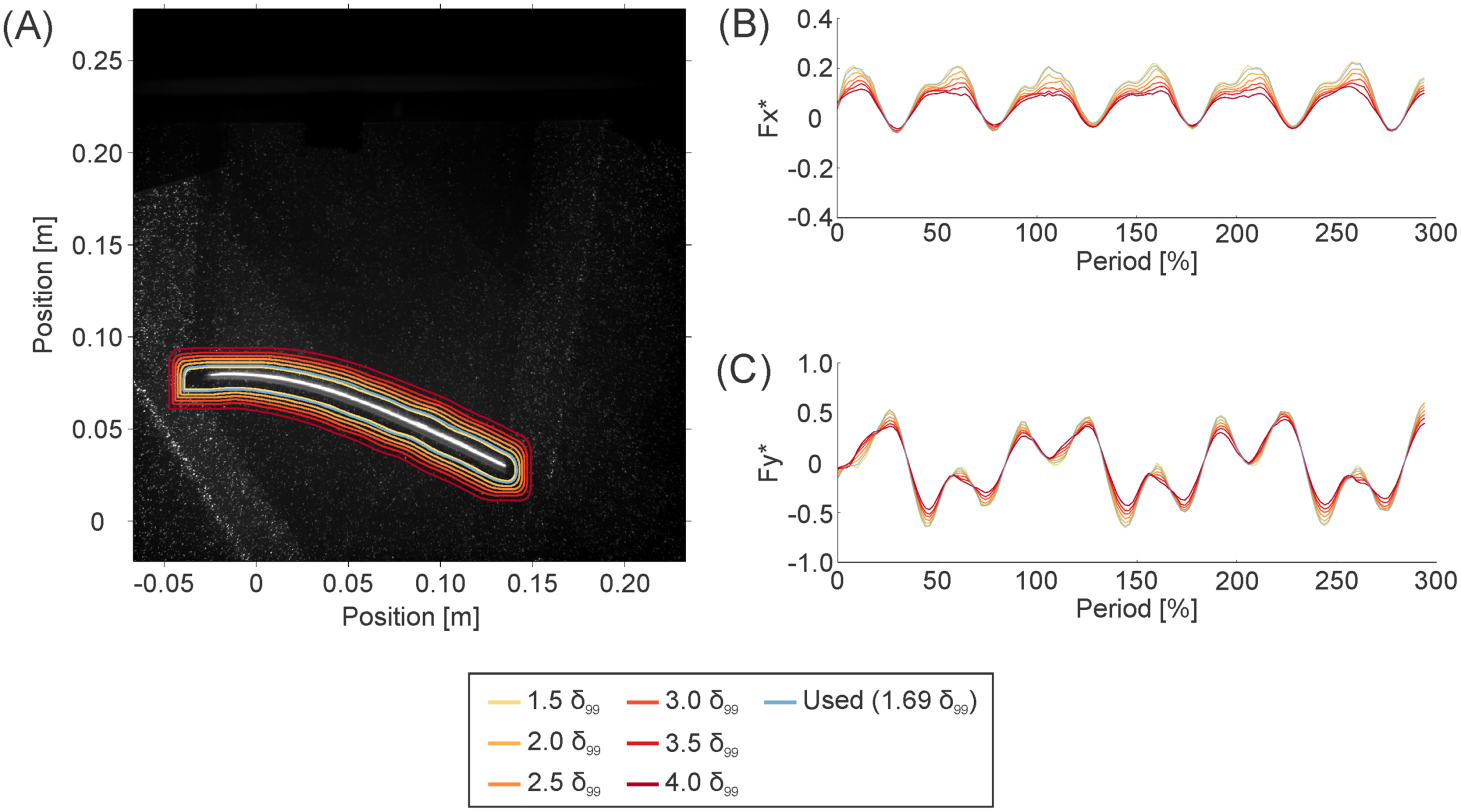
Forces calculated at different boundary positions. The magnitudes of the forces calculated using the pressure-based technique did not decline substantially when the calculation loop was within ~2.5 boundary-layer-widths (*δ*_*99*_) from the foil’s midline. To determine how close to the foil the force calculation loop needed to be for high accuracy results, the pressure-based calculation was conducted on multiple loops around the foil. Tests were conducted using pressure data from the dynamic, 2.0 Hz, 0° angle of attack trial. Loop position was measured in *δ*_*99*_-widths from the foil’s midline. (A) All of the calculation loops examined, drawn on the original image of the foil. (B) Non-dimensional streamwise forces (*F*_x_*) for the different loops, over three periods of foil motion. (C) Non-dimensional lateral forces (*F*_y_*) for the different loops, over three periods of foil motion.

Eqs 1 and 2 were formulated assuming that the control surface—the force calculation loop from the previous paragraph—was drawn at the foil’s surface, but, in practice, this loop must be drawn at a small distance away from the foil. To determine how far away from the foil the control surface could be placed before fluid terms must be added to Eqs 1 and 2 to maintain accuracy, the force calculation process was conducted multiple times using the dynamic test’s 2.0 Hz, 0° angle of attack trial as a test case. In each iteration, the control surface was drawn in a new position. The binary image dilation process was used to place the control surface 1.5, 2.0, 2.5, 3.0, 3.5, and 4.0 *δ*_*99*_ from the foil’s midline (Fig 6A). The resulting *F*_x_* and *F*_y_* time series found in Fig 6B and 6C indicated that limited change to force magnitudes occurred until the surface was placed ~2.5–3.0 *δ*_*99*_ away from the foil’s midline. As such, we chose to draw the final calculation boundaries by dilating the binary image in Fig 4B to a width of 15 pixels, resulting in a loop 1.64 *δ*_*99*_ from the foil’s midline (Fig 6A). The decline observed here suggests that additional fluid terms may be required in Eqs 1 and 2 for accurate force and torque calculation when the calculation boundary is drawn far from the swimmer.

### Metrics for comparing force and torque measurements and estimations

The estimated force and torque values derived from the pressure fields were compared to the measured values from the flapping-foil system to determine their accuracy. Visual inspection could reveal generally how well the two matched, but to quantify the match, three analyses were conducted using a custom Python script. All quantitative comparisons were conducted based on the three successive replicate traces (i.e., not the phased-averaged traces).

First, the correlation coefficient between each pair of corresponding measured and estimated traces was calculated. This would demonstrate how well the shapes of the traces matched. The limits of the 95% confidence interval for each correlation coefficient were calculated to provide a measure of uncertainty in the correlation. The number of significant figures was determined based on the standard error [55].

Next, to determine how well the magnitudes of the two traces matched, root mean square error percentage (RMSE%) was calculated using the equations below, where *M*_*i*_ and *C*_*i*_ represent corresponding force or torque values in the measured trace and calculated trace, respectively.

$$RMSE\%=\frac{\sqrt{\frac{{\sum_{i=1}^{n}\left(M_{i}-C_{i}\right)}^{2}}{n}}}{C_{max}-C_{min}}\times 100=\frac{RMSE}{C_{max}-C_{min}}\times 100$$ (9)

Finally, the presence of any phase lags was revealed using cross-correlation between corresponding measured and estimated time traces. The resulting lags were normalized by the duration of a motion cycle so as to facilitate comparisons.

#### Depositing of data files and scripts

All video and force-torque sensor data files are available from the “Video and sensor data for pressure-based force calculation validation” database on Harvard Dataverse available at http://dx.doi.org/10.7910/DVN/5NCA5X. The Dabiri et al. [35] pressure-field algorithm is freely available at http://dabirilab.com/software/ as executable software in a .p file format, which will launch as a GUI in Matlab where the user can load velocity data and generate the corresponding pressure fields. Please note that the .p file will not render as a readable code in a text editor, and the reader is highly encouraged to reference the ReadMe document provided with the GUI. The algorithm is also available at https://github.com/kelseynlucas, along with all other scripts used for data processing.

## Results

### Sensitivity of force and torque estimation to 3D flows

**Fig 7:**
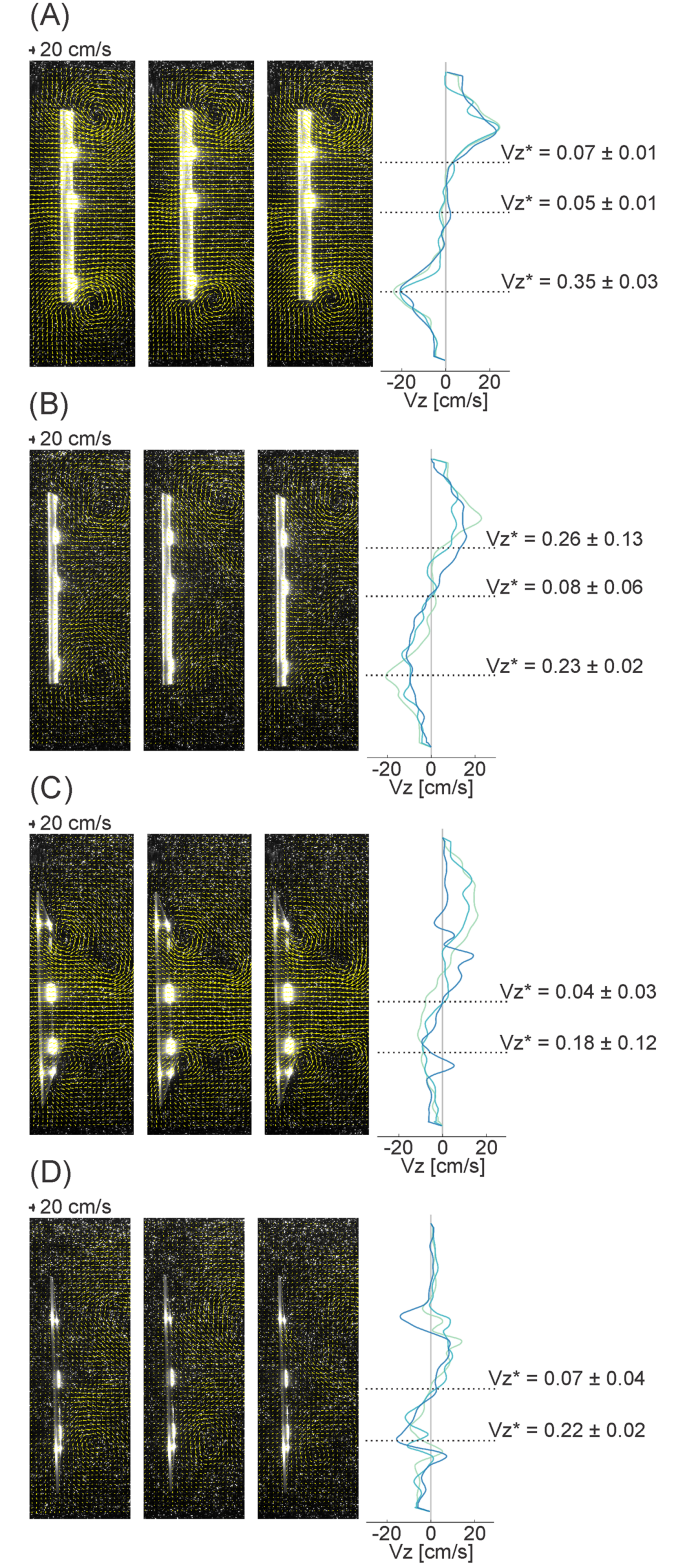
Velocity vector fields in the transverse plane. Velocity vector fields in the transverse plane as the foils approach direction reversal, for 1.5 Hz actuation frequency, 1.5 cm heave amplitude, and 30 cm/s oncoming flow (the conditions used for 3D testing). The foils move toward the left. Vertical velocities (*V*_*z*_) were taken along a vertical line immediately to the right of the foil. Bright spots represent either the edges of the foil or the strips of fluorescent paint. Colors in velocity traces represent different trials. (A) Rectangular foil, 0° angle of attack program. (B) Rectangular foil, heaving program. (C) Tail-shaped foil, 0° angle of attack program. (D) Tail-shaped foil, heaving program. *V*_z_*—*V*_*z*_ normalized by the total velocity at the measurement location, plus or minus standard deviation. Rect—rectangular foil. Tail—tail-shaped foil.

Transverse imaging near the foils’ trailing edges revealed that fluid flow was predominantly in the horizontal plane for both the rectangular and the tail-shaped foils. The proportion, and hence, importance, of vertical flows relative to total flow (*V*_z_*) increased with proximity to the spanwise edges of the foil (Fig 7). The *V*_z_* magnitudes far from the foils’ midlines were highly dependent on the size and strength of tip vortices attached to the spanwise edges of the foils (Fig 7). The largest *V*_z_* observed was 35%, when the rectangular foil was moved in the 0° angle of attack program. Vertical velocity (*V*_*z*_) traces were more complex for the tail-shaped foil than for the rectangular foil, as a result of interactions between the upstream “body” and downstream “tail” portions of this foil. In particular, tip vortices shed from the body portion of the foil upstream are visible on the right side of the flow field in Fig 7C.

**Fig 8:**
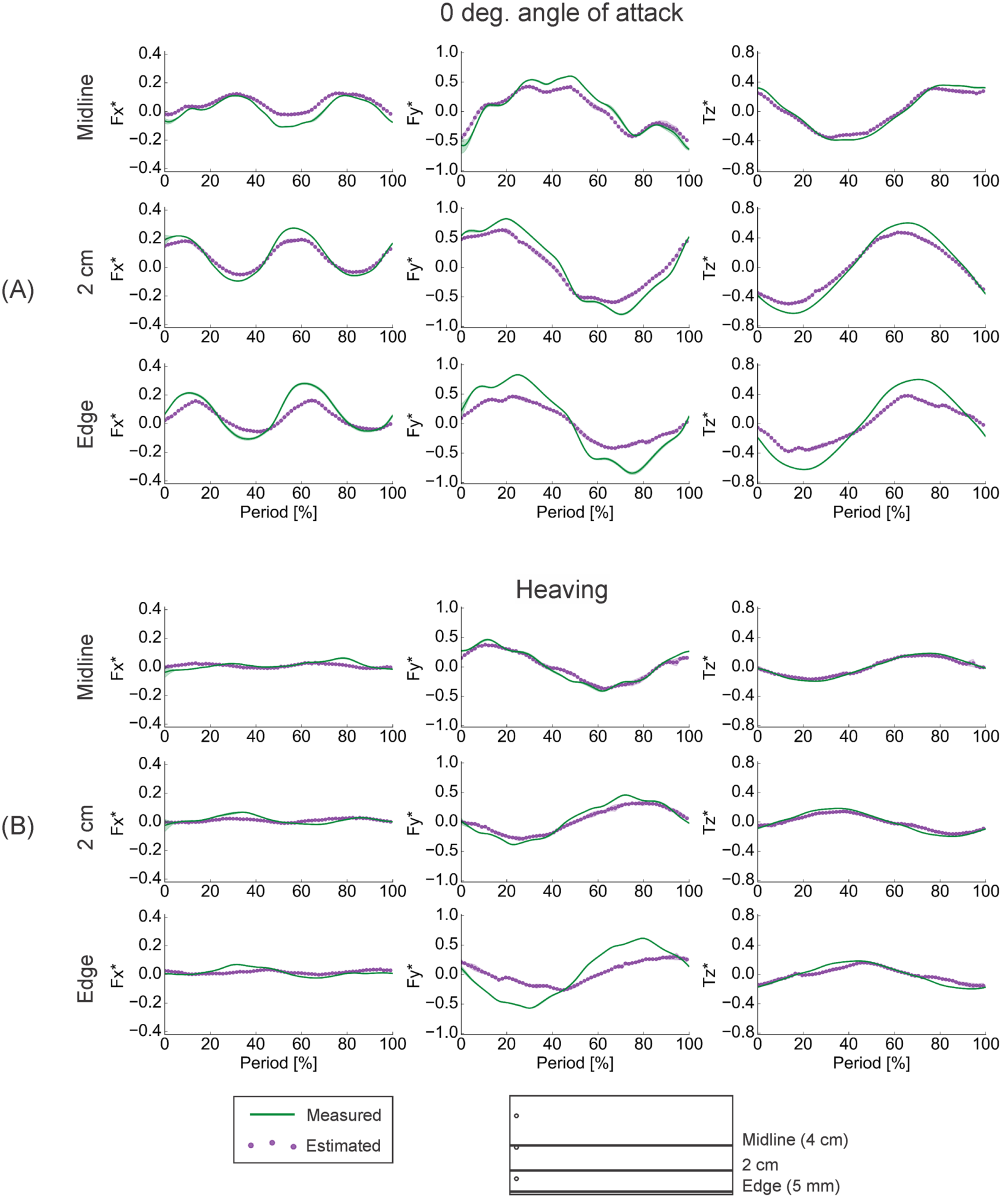
Results of three-dimensional testing of the rectangular foil. Comparisons of phase-averaged (n = 3) measured and calculated force and torque time traces reveal that the pressure-based force calculation was generally able to accurately reproduce both the shape and magnitude of the measured trace, although the agreement declined with proximity to the foil’s edge. (A) Results from 0° angle of attack motions. Due to unusual oscillations resulting from a loose screw, midline data were replaced with equivalent data from dynamic testing. (B) Results from heaving motions. *F*_*x*_ − streamwise forces. *F*_*y*_ − lateral forces. *T*_*z*_ − torques about the vertical axis. Foil kinematics corresponded with the 1.5 Hz cases in dynamic testing (see Figs 10 and 11). Silhouettes represent standard deviations.

**Fig 9:**
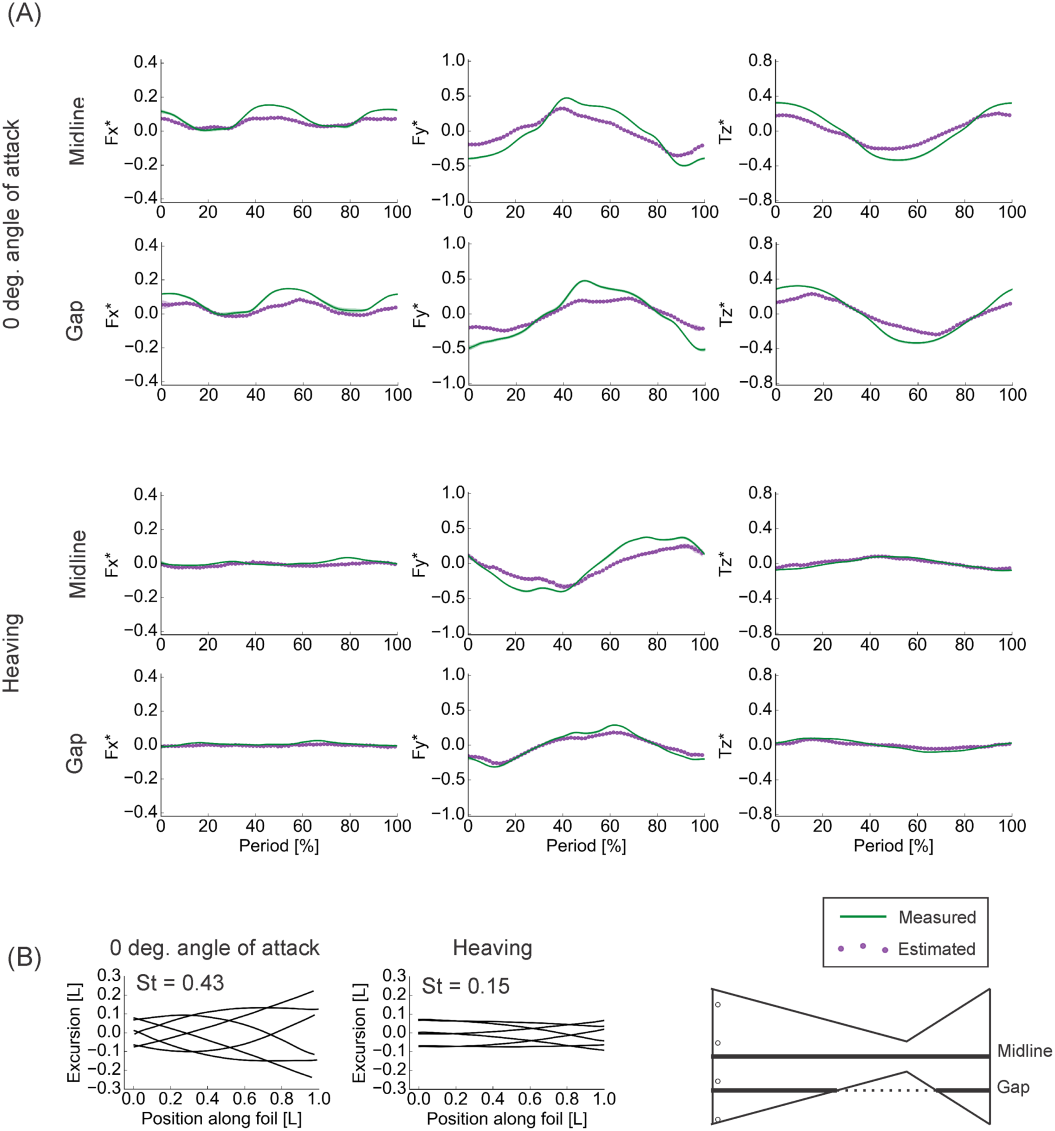
Results of three-dimensional testing of the tail-shaped foil. Despite the more complex shape of this foil, the pressure-based calculations were often able to accurate reproduce both the shape and the magnitude of the measured time traces. (A) Comparisons of phase-averaged (n = 3) measured and calculated time traces. (B) Midline kinematics over one motion cycle. *F*_*x*_ − streamwise forces. *F*_*y*_ − lateral forces. *T*_*z*_ − torques about the vertical axis. St—Strouhal number. Silhouettes represent standard deviations.

During the 3D tests, the agreement between the measured force and torque values from the flapping-foil system and the predictions based on the pressure fields was, in the majority of cases, exceptional. For lateral forces (*F*_*y*_) and torques about the vertical axis (*T*_*z*_), typical correlation coefficients for both foils were greater than 0.9, with typical RMSE% less than 25%, and phase lags less than 5% (Tables 1 and 2). The exceptions to these trends generally were localized to the foils’ spanwise edges, and, as discussed later, these exceptions can point to where the limitations of this method lie. These high, positive correlation coefficients, low RMSE%, and limited phase lags respectively demonstrate that the pressure-based calculation was able to reproduce the shape, magnitude, and timing of the locomotor forces and torques acting on the foils even when the magnitude of vertical flows became more substantial (Figs 8 and 9). Additionally, where the calculated values deviated from the measurements, the calculations tended to underestimate the true values (Figs 8 and 9).

**Table 1:** Rectangular foil—Quantitative comparisons^a^ of forces and torques from three-dimensional tests.

Motion type	Laser position	Correlation Coefficient^b^ (95% CI low, high)			RMSE%			Phase Lag %^c^		
		*F*_*x*_	*F*_*y*_	*T*_*z*_	*F*_*x*_	*F*_*y*_	*T*_*z*_	*F*_*x*_	*F*_*y*_	*T*_*z*_
0angle	Midline^d^	0.963 (0.951, 0.972)	0.937 (0.917, 0.952)	0.9924 (0.9899, 0.9942)	30.3	12.7	7.3	0	1.5	0
	2 cm	0.985 (0.980, 0.989)	0.982 (0.976, 0.986)	0.9972 (0.9963, 0.9979)	15.9	12.2	10.1	0	3	0
	Edge	0.935 (0.915, 0.950)	0.989 (0.985, 0.991)	0.984 (0.978, 0.988)	30.3	30.5	25.4	-1.5	0	-1.5
heave	Midline	0.341 (0.212, 0.459)	0.976 (0.969, 0.982)	0.987 (0.983, 0.990)	45.8	8.2	7.7	9	-1.5	0
	2 cm	0.523 (0.414, 0.617)	0.968 (0.958, 0.976)	0.978 (0.971, 0.983)	51.3	13.4	12.1	-48	-3.0	-1.5
	Edge	0.355 (0.227, 0.471)	0.765 (0.701, 0.818)	0.942 (0.924, 0.956)	55.4	45.2	14.3	-57	-10.5	-1.5

0angle = 0° angle of attack motions; heave = heaving motions; 95% CI = confidence interval; RMSE% = root-mean-square error percentage.

^a^ Note that some quantitative analyses performed poorly at low force magnitudes and may therefore underestimate the agreement between the measured and calculated force and torque values. See Results section in the main text for details.

^b^ Normalized values.

^c^ Phase lags, as percentages of motion cycle periods, were determined by cross-correlation.

^d^ Due to unusual oscillations resulting from a loose screw, midline data were replaced with equivalent data from dynamic testing.

**Table 2:** Tail-shaped foil—Quantitative comparisons^a^ of forces and torques from three-dimensional tests.

Motion type	Laser position	Correlation Coefficient^b^ (95% CI low, high)			RMSE%			Phase Lag %^c^		
		*F*_*x*_	*F*_*y*_	*T*_*z*_	*F*_*x*_	*F*_*y*_	*T*_*z*_	*F*_*x*_	*F*_*y*_	*T*_*z*_
0angle	Midline	0.949 (0.933, 0.961)	0.924 (0.900, 0.942)	0.970 (0.961, 0.977)	50.2	21.8	24.1	0	3	1.5
	Gap	0.887 (0.853, 0.913)	0.963 (0.951, 0.972)	0.969 (0.959, 0.976)	43.9	33.6	20.6	-3	-1.5	-3
heave	Midline	0.424 (0.303, 0.532)	0.924 (0.900, 0.942)	0.932 (0.911, 0.948)	43.0	22.7	12.5	-7.5	-4.5	3
	Gap	0.723 (0.649, 0.783)	0.983 (0.977, 0.987)	0.940 (0.922, 0.955)	36.8	11.9	22.8	0	-1.5	0

0angle = 0° angle of attack motions; heave = heaving motions; 95% CI = confidence interval; RMSE% = root-mean-square error percentage.

^a^ Note that some quantitative analyses performed poorly at low force magnitudes and may therefore underestimate the agreement between the measured and calculated force and torque values. See Results section in the main text for details.

^b^ Normalized values.

^c^ Phase lags, as percentages of motion cycle periods, were determined by cross-correlation.

In all of the 3D tests performed, absolute *F*_*x*_ magnitudes were quite small–<0.2 N, or <0.3 nondimensionalized, compared to <0.8 N or <0.6 nondimensionalized for *F*_*y*_ (Figs 8 and 9), and the pressure-based force calculation was able to reproduce these small magnitudes. Yet, while *F*_*x*_ correlation coefficients were in excess of 0.85 in all but one case in the 0° angle of attack program, the values in the heaving program were about 0.3–0.5 for the rectangular foil and 0.4–0.7 for the tail-shaped foil (Tables 1 and 2). For both foils in both programs, typically, the RMSE% ranged from 15–55%, with phase lags between 0–60% (Tables 1 and 2). To resolve this discrepancy, we must note that because *F*_*x*_ magnitudes were small, slight deviations between the estimated and measured time traces would translate to large percent differences. For this reason, the RMSE% of streamwise forces reported in Tables 1 and 2 are somewhat misleading. Likewise, phase lags hold little meaning when the correlation between two signals is limited.

The misleading nature of the quantitative analyses for small force magnitudes also becomes apparent from the size scales of the 95% confidence intervals for the correlation coefficients (Tables 1 and 2). While the “95% confidence interval” merely means that we are 95% sure that the true correlation coefficient is within the given range, the size of the range can provide a proxy for how uncertain the reported values are. Here, the 95% confidence intervals for the *F*_*x*_ correlation coefficient were 50% to an order of magnitude larger than for those for *F*_*y*_ or *T*_*z*_, particularly where the *F*_*x*_ correlation coefficients were less than 0.8 (Tables 1 and 2). This, again, indicated that some of the quantitative analyses perform poorly where force magnitudes were small. The remainder, however, including the difference in correlation coefficients between 0° angle of attack cases where *F*_x_* < 0.3 nondimensionalized and heaving cases where *F*_x_* < 0.08 nondimensionalized, can reveal some insight into how well this force calculation method will work for studies of biological locomotion, as discussed in later sections.

### Sensitivity of force and torque estimation to flapping frequency

**Fig 10:**
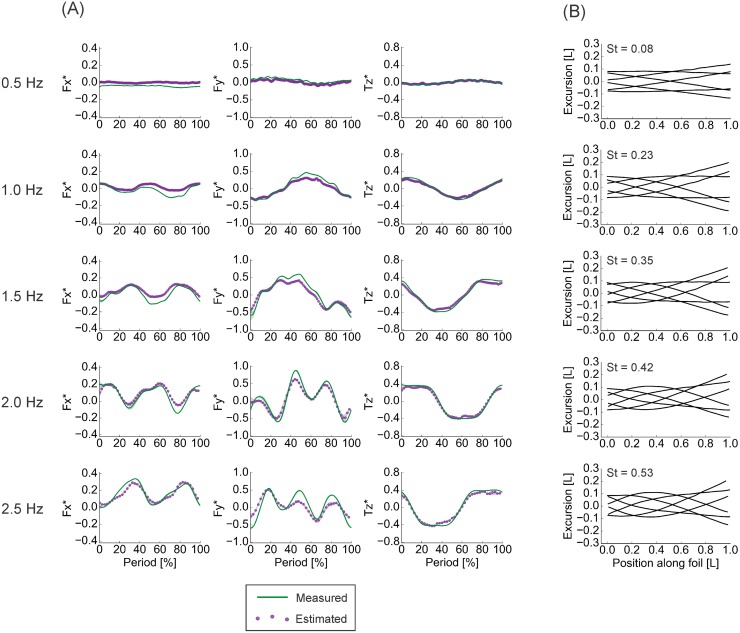
Results of dynamic testing, 0° angle of attack motions, of the rectangular foil. Generally, the pressure-based calculations were able to accurately reproduce both the shape and the magnitude of the measured time traces. Agreement declined slightly as actuation frequency increased. (A) Comparisons of phase-averaged (n = 3) measured and calculated time traces. (B) Midline kinematics over one motion cycle, corresponding to the time traces on the left. *F*_*x*_ − streamwise forces. *F*_*y*_ − lateral forces. *T*_*z*_ − torques about the vertical axis. St—Strouhal number. Silhouettes represent standard deviations.

**Fig 11:**
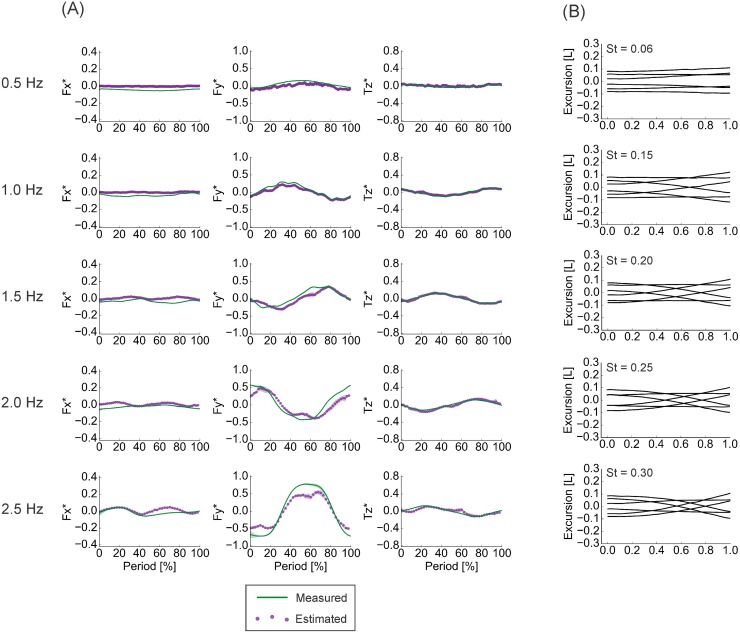
Results of dynamic testing, heaving motions, of the rectangular foil. Generally, the pressure-based calculations were able to accurately reproduce both the shape and the magnitude of the measured time traces, but less so than during 0° angle of attack motions. Agreement declined slightly as actuation frequency increased. (A) Comparisons of phase-averaged (n = 3) measured and calculated time traces. (B) Midline kinematics over one motion cycle, corresponding to the time traces on the left. *F*_*x*_ − streamwise forces. *F*_*y*_ − lateral forces. *T*_*z*_ − torques about the vertical axis. St—Strouhal number. Silhouettes represent standard deviations.

The overall trends resulting from dynamic testing were similar to those from the 3D tests. With one exception, the *F*_*y*_ and *T*_*z*_ correlation coefficients were >0.85. The RMSE% was generally <20%, and phase lags were minimal at <6% (Table 3). The lowest flapping frequency, 0.5 Hz, generally had the poorest agreements among the dynamic tests with slightly smaller correlation coefficients and slightly larger RMSE% and phase lags than the higher frequencies. Again, *F*_*x*_ results were more inconsistent. At all but the lowest frequency, agreements in the 0° angle of attack motion program were better than the heaving program: higher correlation coefficients, lower RMSE%, and smaller phase lags (Table 3). Yet, as in the 3D tests, large 95% confidence intervals (Table 3), a high level of agreement between time-traces in the visualizations (Figs 10 and 11), and low absolute *F*_*x*_ magnitudes (compare <0.5 N or <0.4 nondimensionalized in 0° angle of attack trials to <0.1 N or 0.08 nondimensionalized in the heaving trials) indicated that the quantitative metrics were misleading due to the low performance of the analyses at small force magnitudes. As in the 3D tests, though, these results can still suggest where the limits to the pressure-based calculation method are for the study of biological locomotion, as discussed in later sections.

**Table 3:** Dynamic tests—Quantitative comparison^a^ of forces and torques at increasing actuation frequencies.

Motion type	Freq [Hz]	Correlation Coefficient^b^ (95% CI low, high)			RMSE%			Phase Lag %^c^		
		*F*_*x*_	*F*_*y*_	*T*_*z*_	*F*_*x*_	*F*_*y*_	*T*_*z*_	*F*_*x*_	*F*_*y*_	*T*_*z*_
0angle	0.5	0.248 (0.171, 0.322)	0.908 (0.893, 0.921)	0.914 (0.900, 0.926)	165.5	24.3	10.9	50	0.5	-1.0
	1.0	0.870 (0.840, 0.895)	0.992 (0.990, 0.994)	0.9914 (0.9893, 0.9932)	53.6	13.2	8.0	0	0	0
	1.5	0.963 (0.951, 0.972)	0.972 (0.962, 0.978)	0.9924 (0.9899, 0.9942)	30.3	12.7	7.3	0	1.5	0
	2.0	0.948 (0.929, 0.962)	0.971 (0.960, 0.979)	0.9947 (0.9926, 0.9961)	16.8	10.4	5.9	0	0	0
	2.5	0.929 (0.889, 0.950)	0.792 (0.713, 0.851)	0.9942 (0.9916, 0.9960)	14.7	19.5	6.4	0	0	0
heave	0.5	0.513 (0.451, 0.569)	0.869 (0.848, 0.888)	0.649 (0.601, 0.693)	279.2	28.9	21.7	1.5	0	-1.5
	1.0	0.605 (0.528, 0.672)	0.981 (0.976, 0.985)	0.956 (0.945, 0.965)	165.6	12.4	10.7	2.0	0	-1.0
	1.5	0.152 (0.013, 0.286)	0.883 (0.848, 0.910)	0.956 (0.942, 0.966)	82.0	15.9	8.3	10.5	-6.0	-1.5
	2.0	0.063 (-0.099, 0.222)	0.891 (0.852, 0.920)	0.958 (0.942, 0.969)	57.0	17.5	9.8	-86	-6.0	-2.0
	2.5	0.556 (0.417, 0.669)	0.988 (0.983, 0.992)	0.856 (0.799, 0.898)	36.8	18.3	15.9	2.5	0	-5.0

0angle = 0° angle of attack motions; heave = heaving motions; Freq = frequency; 95% CI = confidence interval; RMSE% = root-mean-square error percentage.

^a^ Note that some quantitative analyses performed poorly at low force magnitudes and may therefore underestimate the agreement between the measured and calculated force and torque values. See Results section in the main text for details.

^b^ Normalized values.

^c^ Phase lags, as percentages of motion cycle periods, were determined by cross-correlation.

The RMSE% values, alongside the visualizations in Figs 10 and 11, indicated that the agreement between the direct measurements and pressure-based estimates was best at moderate flapping frequencies and poorest at the extremes. These moderate frequencies are close to the frequencies where the rectangular foil achieves self-propelled speed—the speed where net forces and torques over a motion cycle are zero (occurs at 1.0 Hz during 0° angle of attack motions, 1.5 Hz during heaving motions) [11]. Even so, the generally high level of agreement indicated that this method of pressure-based estimation of locomotor forces and torques will perform well for a swimmer in steady, accelerating, and deaccelerating motion, provided the swimmer is not moving particularly slowly—e.g., the 0.5 Hz flapping frequency case, which is at the lower extreme of tailbeat frequencies used by fish [16,48,49].

### Results from static testing

**Fig 12:**
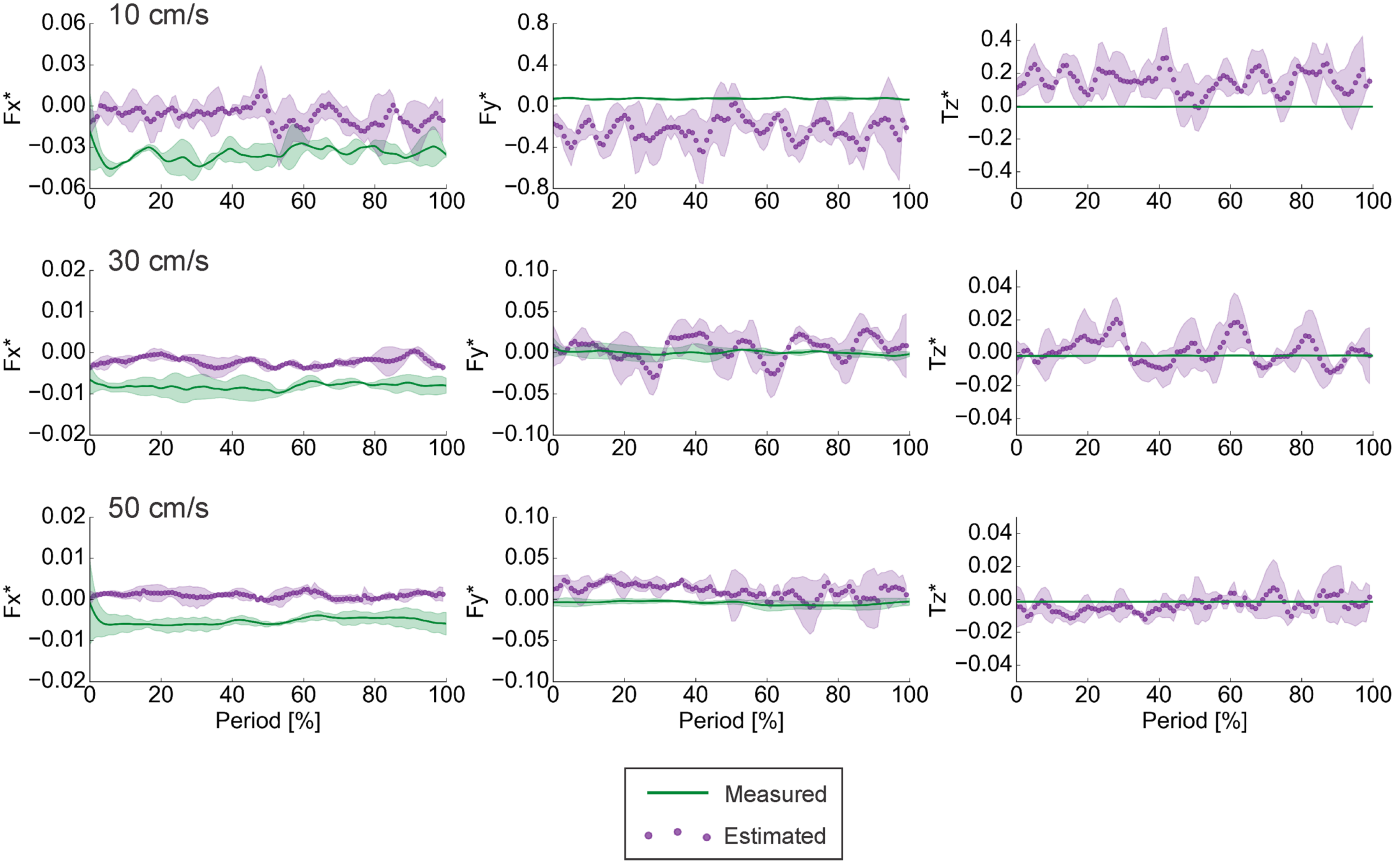
Results of static testing of the rectangular foil. In the static case, where shear forces dominate, the pressure-based calculations were not able to accurately predict locomotor forces and torques. For the comparisons of phase-averaged (n = 3) measured and calculated time traces, note that the y-axis scales vary among oncoming flow speeds and differ from those in other figures. *F*_*x*_ − streamwise forces. *F*_*y*_ − lateral forces. *T*_*z*_ − torques about the vertical axis. Silhouettes represent standard deviations.

As anticipated, when the foil was held statically and viscous effects were relatively more important than pressure effects, the pressure-based estimates of forces and torques were very poor (Fig 12). Correlation coefficients clustered around zero, and there was no consistent trend in RMSE% (Table 4). In particular, the pressure-based calculation was unable to detect any drag on the foil, though the measurements consistently revealed negative *F*_*x*_ (Fig 12).

**Table 4:** Static tests—Quantitative comparison of forces and torques at increasing oncoming flow speeds.

Flow speed [cm/s]	Correlation Coefficient^a^ (95%CI low, high)			RMSE%		
	*F*_*x*_	*F*_*y*_	*T*_*z*_	*F*_*x*_	*F*_*y*_	*T*_*z*_
10	-0.117 (-0.227, -0.003)	-0.132 (-0.242, -0.018)	-0.016 (-0.129, 0.098)	44.6	34.6	29.9
30	0.080 (-0.034, 0.192)	0.127 (0.014, 0.237)	0.170 (0.057, 0.278)	82.8	19.7	19.6
50	0.001 (-0.112, 0.115)	0.588 (0.508, 0.658)	0.278 (0.170, 0.379)	98.0	27.1	19.5

95% CI = confidence interval; RMSE% = root-mean-square error percentage.

^a^ Normalized values.

## Discussion

Many of the diverse morphological features of fishes are tied to swimming behaviors, and consequently, understanding a fish’s locomotion is an integral part of answering many unresolved questions about evolution and function in fishes. The ability to use standard 2D DPIV data to calculate the instantaneous distribution of forces and torques on freely-swimming fishes would represent a substantial advance in our ability to study aquatic locomotion. Hence, we propose a pressure-based method for obtaining this information non-invasively and focus here on validating this approach and determining the experimental conditions under which it works best. Our comparisons of the forces and torques estimated through pressure-based calculations to the values measured by a load cell revealed that, under many conditions, the pressure-based calculation was able to accurately estimate time-dependent locomotor forces and torques. We were often able to reproduce the shape, magnitude, and timing of the measured traces.

The points at which the pressure-based calculations led to low accuracy force and torque estimations suggest where the limits of this approach for biological study lie. Largely, the key factors responsible for the method’s performance were based on the validity of the assumptions behind the technique: 1) that the fluid velocity perpendicular to the horizontal imaging plane is relatively small, an assumption inherent to 2D DPIV [42], and 2) that the Reynolds number is sufficiently high such that the pressure (inertial) term in Eqs 1 and 2 dominates the shear (viscous) term. In the following sections, we leverage our data on the relative roles of horizontal vs. out-of-plane flows and the pressure vs. shear effects to explain where the limits of this pressure-based approach to force and torque calculation lie for biological locomotion studies. We find that where a given trial falls on each of these spectra together determines the accuracy of the calculation.

Notably, the agreement between measured and estimated force and torque values was best during 0° angle of attack motions, a kinematic regime more similar to the motions used by a swimming fish [11,15]. Moreover, the agreement for streamwise—thrust or drag—forces was at its best in this program at higher flapping frequencies (Figs 10 and 11; Table 3), which correspond more closely to typical tailbeat frequencies used by fish [16,48,49], and additionally, the agreement was strong close to the foils’ spanwise edges (Figs 8 and 9; Tables 1 and 2). This points to the especial utility and promise of this force and torque estimation procedure for studies of biological swimming.

### Limitations due to 2D methodology

As a whole, the effectiveness of the pressure-based force and torque estimation using the first terms in Eqs 1 and 2 was highly dependent on the quality of the DPIV data. While factors such as temporal and spatial resolution and DPIV uncertainty do affect the outcome, their effects can easily be addressed using good imaging technique—e.g., those outlined by Stamhuis and Videler [42] and de Kat and van Oudheusen [56]–and post-hoc smoothing of velocity fields [52]. Less apparent, however, are the limitations posed by using a 2D plane to characterize a flow through a 3D space. Yet, this approach is often the most feasible option in biological studies that measure locomotor flows.

While flows around a real, 3D object will inherently have some degree of three-dimensionality, in many cases, a 2D analysis can provide sufficient information to answer the questions posed in a given experiment—for example, Drucker and Lauder [17]. How we decide whether 2D analysis is sufficient is, to some extent, subjective, but the 3D tests on the rectangular and tail-shaped foils demonstrate that some degree of deviation from the 2D assumption will still lead to fairly accurate results (Figs 8 and 9).

We found that proximity to the edge of the rectangular foil, where the relative magnitude of vertical flows (*V*_z_*) was greatest, was associated with poorer force and torque predictions (Fig 7; Table 1). The pattern of the decline did not vary substantially between motion programs (Table 1). The exception was lateral force (*F*_*y*_) at this foil’s edge, which saw a large increase in error only in the heaving program (Table 1). It is likely that the sweep of fluid around the edge of the foil as it heaved laterally (Fig 7) reduced the build-up of pressure gradients near the edge, underestimating the gradients at other locations along the foil’s span. This effect on *F*_*y*_ would be more profound in the heaving program where the majority of the foil’s surface area faces laterally (Fig 11B). Thus, the pressure-based forces also primarily point laterally.

**Fig 13:**
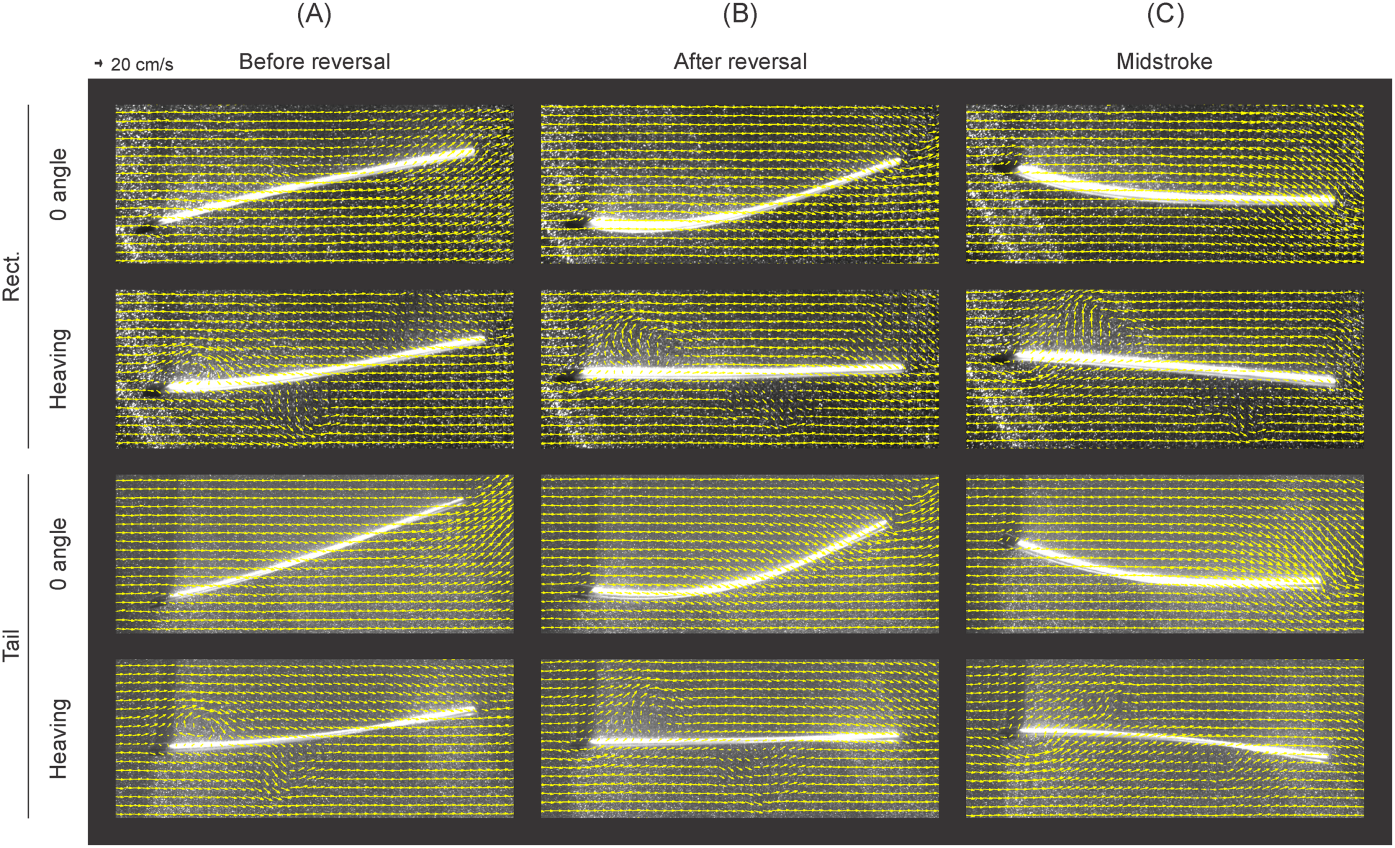
Velocity vector fields in the horizontal plane, at the foil’s midline. Velocity vector fields at the foils’ midlines revealing the differences in flow structures around the rectangular (Rect) and tail-shaped (Tail) foils during 0° angle of attack and pure heaving motions at three points in a stroke cycle. The foils were moved at 1.5 Hz actuation frequency and 1.5 cm heave amplitude, in an oncoming flow of 30 cm/s. Before direction reversal, the foils move downward. While flow moves smoothly along the foils during 0° angle of attack motions, in the heaving program, a leading edge vortex is formed, shed, and destroyed in the succession of images.

**Fig 14:**
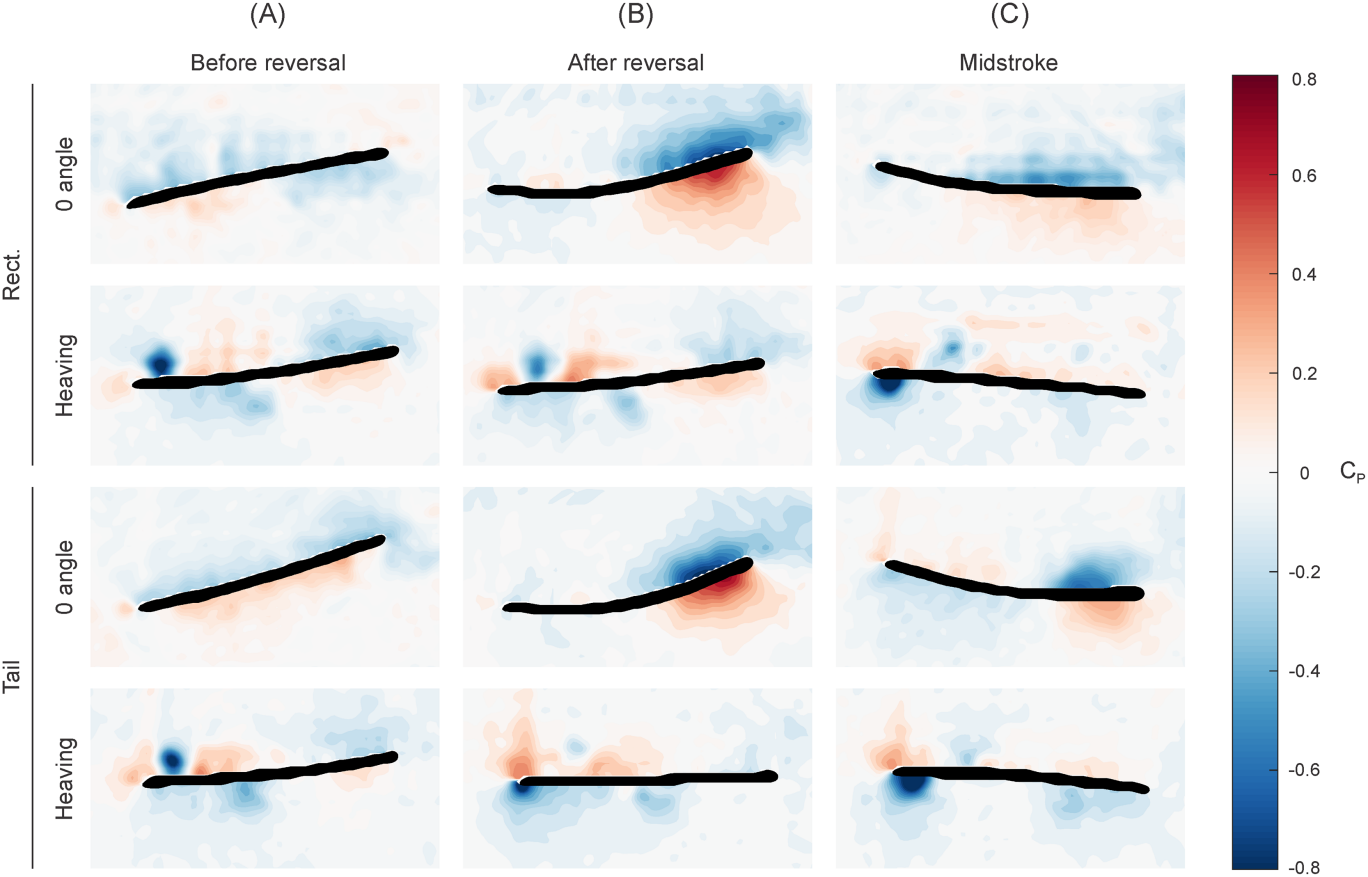
Pressure fields in the horizontal plane, at the foil’s midline. Pressure fields at the foils’ midlines around the rectangular (Rect) and tail-shaped (Tail) foils during 0° angle of attack and pure heaving motions at three points in a stroke cycle, corresponding to the snapshots in Fig 13. The foils were moved at 1.5 Hz actuation frequency and 1.5 cm heave amplitude, in an oncoming flow of 30 cm/s. Before direction reversal, the foils move downward. Color bar indicates the coefficient of pressure (C_P_). During 0° angle of attack motions, pressure gradients peak near the trailing edge. In the heaving program, pressure peaks in the leading edge vortex, and deteriorates into complex patterns as the vortex is impacted by the foil.

Another key observation from the rectangular foil is that the agreement between estimated and measured force and torque traces was generally better during the 0° angle of attack than during the heaving program (Table 1). The explanation is straightforward. When this foil is moved in heave, a strong leading edge vortex forms, leading to a low pressure peak (Figs 13 and 14). During direction reversal, this vortex is shed and begins to travel downstream, and is subsequently impacted by the foil (Figs 13 and 14). The resulting complex flow persists through the next motion cycle and affects approximately the first 40% of the foil’s length (Figs 13 and 14), and likely has significant vertical components. While this does lead to large pressure magnitudes in the horizontal plane (Fig 14), the 2D visualization does not capture the vertical effects, leading to underestimates of forces and torques. It is worth noting that these transient but potentially highly 3D flows also affect estimations at the highest flapping frequencies studied, where direction reversals, and hence, vortex impacts, occur more often. This is apparent in the slight decline in estimation accuracy during these tests (Figs 10 and 11; Table 3). In contrast, the poor agreements at low frequencies can be attributed to shear, as discussed in later sections.

Unlike the heaving program, the 0° angle of attack motion sweeps fluid smoothly and accelerates it along the foil’s length without forming a leading edge vortex or pressure peak (Figs 13 and 14). This smooth flow and acceleration indicates that the tip vortices are entraining fluid and increasing in strength along the entirety of the foil’s length before being shed near the trailing edge, rather than being distorted through interaction with leading edge vortices. Thus, for the rectangular foil in the 0° angle of attack program, the relative contribution of out-of-plane flows (*V*_z_*) measured near the foil’s trailing edge (Fig 7) represent maximums. The limited importance of vertical flows in this program (<35%) ensures that the 2D assumptions are valid, and so the estimation is successful.

The trends of force estimation success were somewhat different for the tail-shaped foil (Fig 9; Table 2). At the foil’s midline, the estimation was marginally better in the 0° angle of attack program, as observed in the rectangular foil. But, when the laser was positioned to cross the foil’s gap, the results were approximately the same in the two motion programs.

The flow visualizations (Figs 7, 13 and 14) shed some light—the more complex foil shape led to more complicated fluid interactions. This foil’s shape, particularly the narrowing of the body into the peduncle, entrains fluid into tip vortices that are angled up or down, following the body, rather than aligned horizontally as in the rectangular foil (Fig 7). The resulting large vertical effects are compounded by the 0° angle of attack kinematics, where the foil sweeps through greater lateral excursions (Fig 9B) [11,15], which further accelerates fluid along the foil (Fig 13). This greatly strengthens the tip vortices attached to the foil’s body and amplifies the vertical flows, until these vortices are shed anterior to the peduncle. These shed vortices are visible on the right side of Fig 7C, and appear to interact with the new tip vortices developing on the foil’s tail region. When the horizontal laser was positioned so as to cross the gap in the foil, the light sheet intersected both these complex flows in the tail region and the strong vertical flows in the body region (Figs 7, 13 and 14), leading to a reduction in accuracy of the force estimation. Note that, as the transverse light sheet was positioned posterior to the peduncle, the *V*_z_* values in Fig 7C and 7D are underestimates of the vertical velocities on the foil as a whole. In contrast, the foil experiences less lateral excursion in the heaving program (Fig 9B) and weaker tip vortices, which are not shed or have dissipated upstream of the transverse light sheet position (Fig 7D), so the relative importance of vertical flows—and the deviation from the 2D assumption—is minimal. Thus, when the horizontal laser is positioned at the gap in the heaving program, the force estimation accuracy does not decline.

Generally, though, we observed high correlation, limited phase shifting, and low error percentage (Tables 1 and 2), as well as the reproduction of general trends such as increasing forces and torques with flapping frequency (Figs 10 and 11). These observations indicate that, where the 2D assumption behind DPIV is valid—e.g., vertical flows are less than ~30% of the total velocity magnitude at any given point (Fig 7)–the 2D pressure-based calculation will be sufficient to provide reasonable estimates of locomotor forces and torques. It is likely that this will also be true in the event of slightly larger *V*_z_*’s, which may be experienced in the anterior 50% of the rectangular foil during heaving motions and of the tail-shaped foil during 0° angle of attack motions, but were not captured at the given transverse light sheet position.

### Role of pressure vs shear effects

Pressure-based force and torque calculations rely on the assumption that the shear terms in Eqs 1 and 2 are small relative to the pressure terms, and hence, the shear terms can be ignored without greatly affecting the accuracy of the result. This assumption is typically met for fish-like swimmers operating at high enough Re. Moreover, Bale et al. [29] suggest that the pressure and shear terms are inversely related: when one is large, the other is small. We illustrate this tradeoff in an extreme with data from the static case. Here, pressure effects (first term in Eqs 1 and 2) are minimal and shear effects (second term in Eqs 1 and 2) dominate: pressure-based calculations alone are unable to estimate the forces and torques experienced by the foil (Fig 12; Table 4).

The tradeoff between shear and pressure effects, however, implies that a middle ground exists where both effects moderately contribute to the total forces. Here, the values calculated from the first terms in Eqs 1 and 2 might provide a good, but not perfect, estimation of true forces and torques. The question is, where does this middle ground fall during fish-like locomotion?

The dynamic test results offer some insight on this point. Agreement between the measurements and the pressure-based estimations improved with flapping frequency (until the 3D effects described in the previous section caused a decline), and was better in the 0° angle of attack program than the heaving program (Figs 10 and 11; Table 3).

While the large errors in *F*_*x*_ were in part due to the small absolute force magnitudes—small magnitude deviations therefore translated to large percent changes—the relatively low level of agreement in *F*_*x*_ versus the higher level of agreement for *F*_*y*_ and *T*_*z*_ suggest that the accuracy of estimation is dependent on the relative proportion of pressure versus shear effects on a given axis.

Two key facts lead to this idea: first, pressure forces act normal to a surface, and second, the majority of the surface area of our foil models—essentially flat plates—faces laterally (Figs 9B, 10B and 11B), normal or nearly normal to the axis of progression during swimming. Thus, on the lateral axis, the large pressure-based contribution dominates the shear effects, so the force estimation performs well. In contrast, the much smaller surface area facing in the streamwise direction leads to a small pressure-based contribution that cannot dominate the shear forces. Instead, the shear term in Eq 1 is relatively large, and so a purely pressure-based force calculation yields an underestimate of *F*_*x*_. This effect is magnified as less surface area is aligned axially, as in motions where the foil experiences limited bending—i.e., heaving motions, and at low frequencies (Figs 10B and 11B). The static foil cases (Fig 12), moreover, could be considered an extreme example of this effect and show some of the poorest *F*_*x*_ predictions. At the other extreme are the results for the dynamic test at 2.5 Hz, the highest frequency tested, in the 0° angle of attack program (Fig 10). The high degree of foil bending here leads to the largest streamwise-facing surface area, and the best *F*_*x*_ and poorest (relatively speaking; the correlation coefficient was 0.792) *F*_*y*_ estimates observed (Fig 10; Table 3).

Unlike flat plates, biological swimmers have greater thickness, and hence, more axial surface area. In addition, head oscillation, common in swimming fishes [7,57], results in a substantial contribution to the streamwise-facing surface area. Fish also tend to follow a kinematic regime more similar to the 0° angle of attack program [11,15], which increases bending (Figs 10B and 11B), and again, axially-oriented surface area. Thus, while we would predict that the relative proportion of surface area can provide an idea of how well pressure-based force estimation will perform on a given axis, it is likely that for many biological swimmers, this pressure-based force and torque estimation method will provide reasonable values in both the lateral and streamwise directions.

### Implications for fish locomotion studies

Together, the 3D and dynamic tests illustrate how 1) the relative importance pressure versus shear and 2) the 3D effects both are factors affecting the accuracy of the force and torque calculations. Moreover, as we have described in the proceeding sections, these two factors appear to have opposing effects relative to the actuation frequency. That is, the maximum frequency (2.5 Hz) and the minimum frequency (0.5 Hz) both led to less accurate outcomes than the moderate frequencies in between. Yet, the decline in performance at the lowest frequency due to shear was much more substantial than the impact of 3D flows at the highest frequency. This is consistent with the main assumption behind our method of force and torque calculation—the assumption that allowed the simplification of Eqs 1 and 2, namely, that the pressure effects are large relative to the shear effects.

Notably, the pure heaving motions are less biologically realistic [11,15] than the 0° angle of attack motions which also increase bending along the foil’s length and the effective axial surface area. The 0° angle of attack motions additionally eliminate the vortex impacts that led to substantial out-of-plane flow in the heaving program. The lowest frequencies tested here, while within the range of tailbeat frequencies used by fish [16,48,49], correspond to the lower extreme of this range. Thus, it appears that, during typical fish-like locomotion, represented here by higher frequencies and 0° angle of attack motions, the pressure effects dominate the shear effects and the 3D effects are sufficiently small, and so the pressure-based estimation of locomotor forces and torques will perform well.

## Conclusion

Here, we described an experimental method for obtaining time-varying swimming force and torque data using standard 2D DPIV in conjunction with a pressure field algorithm. Using a mechanical flapping foil apparatus that models fish-like swimming and simultaneous DPIV video collection and subsequent calculations, we characterized the conditions where this approach is expected to work well. We demonstrated that this technique is often able to accurately reproduce the shape, magnitude, and timing of locomotor forces and torques experienced by a fish-like swimmer.

Detailed knowledge of the time-varying forces and torques acting on a fish’s body is a key component of answering many unresolved questions about form and function in fish, but these data are difficult to measure with the necessary detail and while allowing the animal to swim freely. Our results indicate that pressure-based methods such as that studied here can readily provide the missing detailed, instantaneous force and torque information that, in the past, precluded a more comprehensive understanding of biological swimming.
